# Genetic Drift Versus Climate Region Spreading Dynamics of COVID-19

**DOI:** 10.3389/fgene.2021.663371

**Published:** 2021-12-23

**Authors:** R. Di Pietro, M. Basile, L. Antolini, S. Alberti

**Affiliations:** ^1^ Department of Medicine and Aging Sciences, Section of Biomorphology, G. d’Annunzio University of Chieti-Pescara, Chieti, Italy; ^2^ Center for Biostatistics, Department of Clinical Medicine, Prevention and Biotechnology, University of Milano-Bicocca, Monza, Italy; ^3^ Unit of Medical Genetics, Department of Biomedical Sciences - BIOMORF, University of Messina, Messina, Italy

**Keywords:** COVID-19, pandemic, spreading dynamics, mutation rates, propagation model

## Abstract

**Background:** The current propagation models of COVID-19 are poorly consistent with existing epidemiological data and with evidence that the SARS-CoV-2 genome is mutating, for potential aggressive evolution of the disease.

**Objectives:** We looked for fundamental variables that were missing from current analyses. Among them were regional climate heterogeneity, viral evolution processes versus founder effects, and large-scale virus containment measures.

**Methods:** We challenged regional versus genetic evolution models of COVID-19 at a whole-population level, over 168,089 laboratory-confirmed SARS-CoV-2 infection cases in Italy, Spain, and Scandinavia at early time-points of the pandemic. Diffusion data in Germany, France, and the United Kingdom provided a validation dataset of 210,239 additional cases.

**Results:** Mean doubling time of COVID-19 cases was 6.63 days in Northern versus 5.38 days in Southern Italy. Spain extended this trend of faster diffusion in Southern Europe, with a doubling time of 4.2 days. Slower doubling times were observed in Sweden (9.4 days), Finland (10.8 days), and Norway (12.95 days). COVID-19 doubling time in Germany (7.0 days), France (7.5 days), and the United Kingdom (7.2 days) supported the North/South gradient model. Clusters of SARS-CoV-2 mutations upon sequential diffusion were not found to clearly correlate with regional distribution dynamics.

**Conclusion:** Acquisition of mutations upon SARS-CoV-2 spreading failed to explain regional diffusion heterogeneity at early pandemic times. Our findings indicate that COVID-19 transmission rates are rather associated with a sharp North/South climate gradient, with faster spreading in Southern regions. Thus, warmer climate conditions may not limit SARS-CoV-2 infectivity. Very cold regions may be better spared by recurrent courses of SARS-CoV-2 infection.

## Introduction

Studies on early dynamics of COVID-19 (Li et al., 2020a) revealed that the epidemic doubled in size every 6.4 (Wu et al., 2020) to 7.4 (Li et al., 2020a) days, with a reproductive number (R_0_) of infectious cases of 2.2 (Li et al., 2020a) to 2.7 (Wu et al., 2020). Later investigations followed disease spreading to Singapore (Pung et al., 2020), Germany (Hoehl et al., 2020), France, and Finland (www.ecdc.europa.eu/en/covid-19-pandemic) (Lipsitch et al., 2020; McMichael et al., 2020; Sun et al., 2020). However, major uncertainties remained on SARS-CoV-2 transmission dynamics (Lipsitch et al., 2020). Considerable effort across major research institutions was invested into modeling SARS-CoV-2 spreading determinants. Models were generated, which took into account, among others, global traveling, population density, demographic characteristics, age distribution, social dynamics, governmental policies, air pollution, virus infectious capacity, and SARS-CoV-2 containment procedures, together with economical and healthcare factors (Baker et al., 2020; Hernandez-Vargas and Velasco-Hernandez, 2020; Kennedy et al., 2020; Kissler et al., 2020; Lipsitch et al., 2020; Pearson et al., 2020) (10.21203/rs.3.rs-82122/v1). However, limited, if any, regional heterogeneity in COVID-19 transmission could be identified using such diffusion models. We reasoned that fundamental variables were missing from current analyses, and we went on to identify such missing factor(s).

**FIGURE 1:**
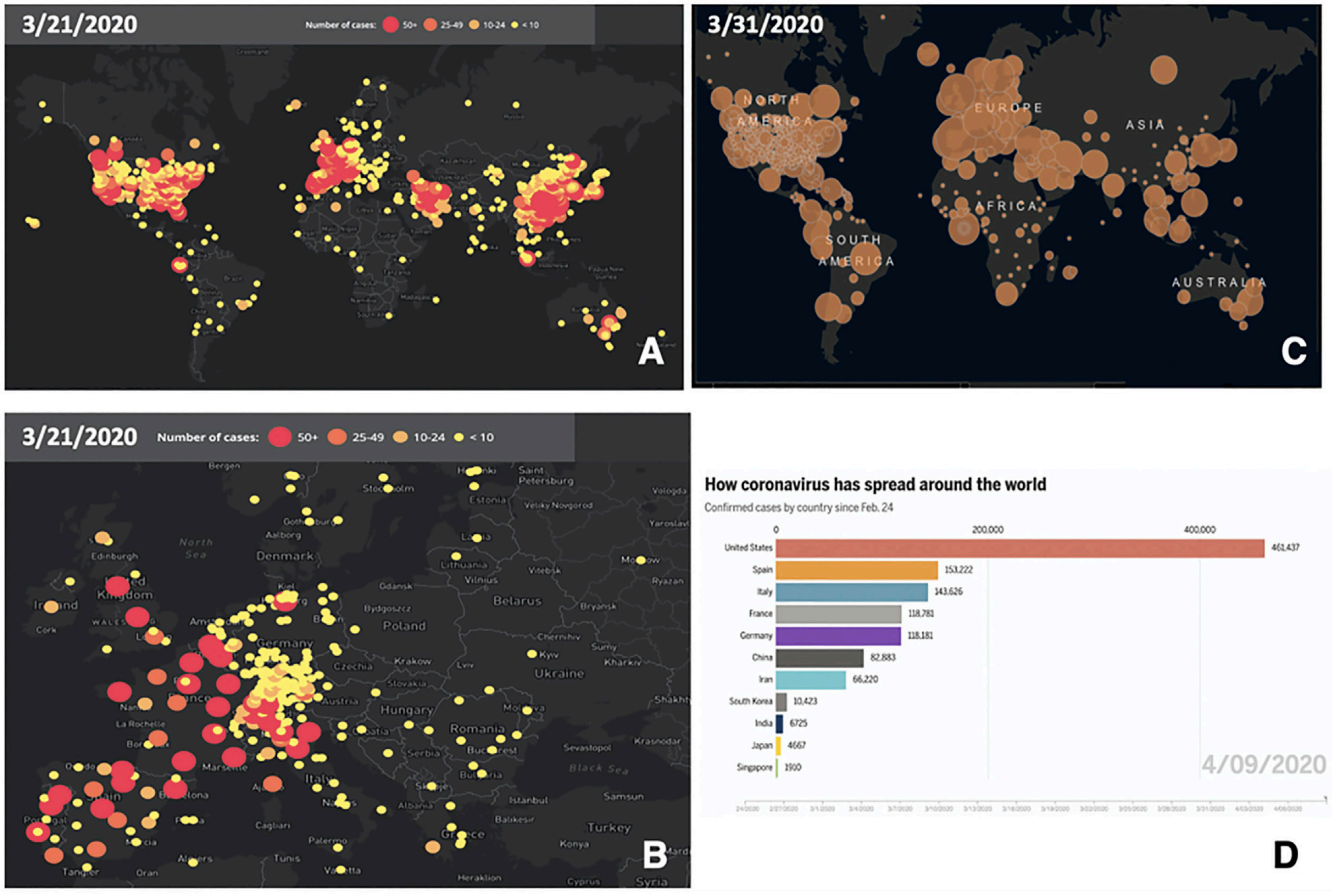
Worldwide progression of COVID-19 cases. **(A)** COVID-19 cumulative case incidence across the world, as of March 21, 2020; numbers are color-coded and are proportional to circle diameter (www.healthmap.org/covid-19/). **(B)** COVID-19 cumulative case incidence, as in (**A**), zoomed over Central Europe. **(C)** COVID-19 incidence of active cases, as of March 31, 2020; numbers are proportional to circle diameter (Johns Hopkins University, JHU; coronavirus.jhu.edu/map.html). **(D)** Coronavirus spreading around the world as of April 9, 2020. Overall confirmed cases by country since February 24, 2020 (JHU, public.flourish.studio/visualisation/1694807/).

SARS-CoV-2 was suggested to be sensitive to temperature and humidity, which may affect diffusion across diverse climate areas (Sundell et al., 2016) (papers.ssrn.com/sol3/papers.cfm?abstract_id=3550308; ssrn.com/abstract=3556998; www.medrxiv.org/content/10.1101/2020.02.22.20025791v1). Accordingly, initial climate-dependent propagation models predicted a limited impact of COVID-19 in the Southern hemisphere, during seasons that were infection-prone in the Northern hemisphere (papers.ssrn.com/sol3/papers.cfm?abstract_id=3550308; ssrn.com/abstract=3556998). However, early foci of infection were detected in Australia and New Zealand (Figure 1). Outbreaks were also revealed in South America and extended to Central America and Mexico. Further infection foci were revealed in Saudi Arabia and Africa and extended to sub-Saharan countries (Supplementary Tables S1, S2), questioning simple models of cold-climate-dependent SARS-CoV-2 transmission.

Viral evolution processes (Eletreby et al., 2020) may mimic regional COVID-19 spreading dynamics (Rausch et al., 2020; Volz et al., 2021; Korber et al., 2020; Li et al., 2020b). SARS-CoV-2 possesses a single-strand RNA genome (Mousavizadeh and Ghasemi, 2020), prone to acquire genomic mutations (nextstrain.org/ncov/; www.gisaid.org/). However, the SARS-CoV-2 RNA polymerase has error-correcting capacity and shows replication error rates >10-fold lower than other RNA viruses (Rausch et al., 2020). Correspondingly, SARS-CoV-2 overall sequence diversity is low (Korber et al., 2020). Spike proteins in particular showed few overall mutations at early time points of the pandemic (Korber et al., 2020). Still, SARS-CoV-2-bearing distinct sets of mutations were isolated in different regions of the world (nextstrain.org/ncov/global), leaving the question open as to whether viral genetic drift, driving distinct disease evolution, could account for heterogeneous disease courses across different geographic areas.

We thus went on to challenge regional versus genetic evolution models of COVID-19 at a population-wide level. The best chances for detecting basic transmission determinants of SARS-CoV-2 were expected before any large-scale defensive approach was implemented (Carleton et al., 2021). Western Europe provided a vast terrain for this approach, because of the large-scale outbreaks of COVID-19 early-on during the pandemic. A further advantage was provided by Europe’s high healthcare management and data collection standards (Bloomberg Global Health Index, 2018, www.bloomberg.com/; WHO, www.who.int/whr/en/; worldpopulationreview.com/countries/best-healthcare-in-the-world/; (Lozano et al., 2020)), which supported a robust detection of basic diffusion parameters of COVID-19.

**FIGURE 2:**
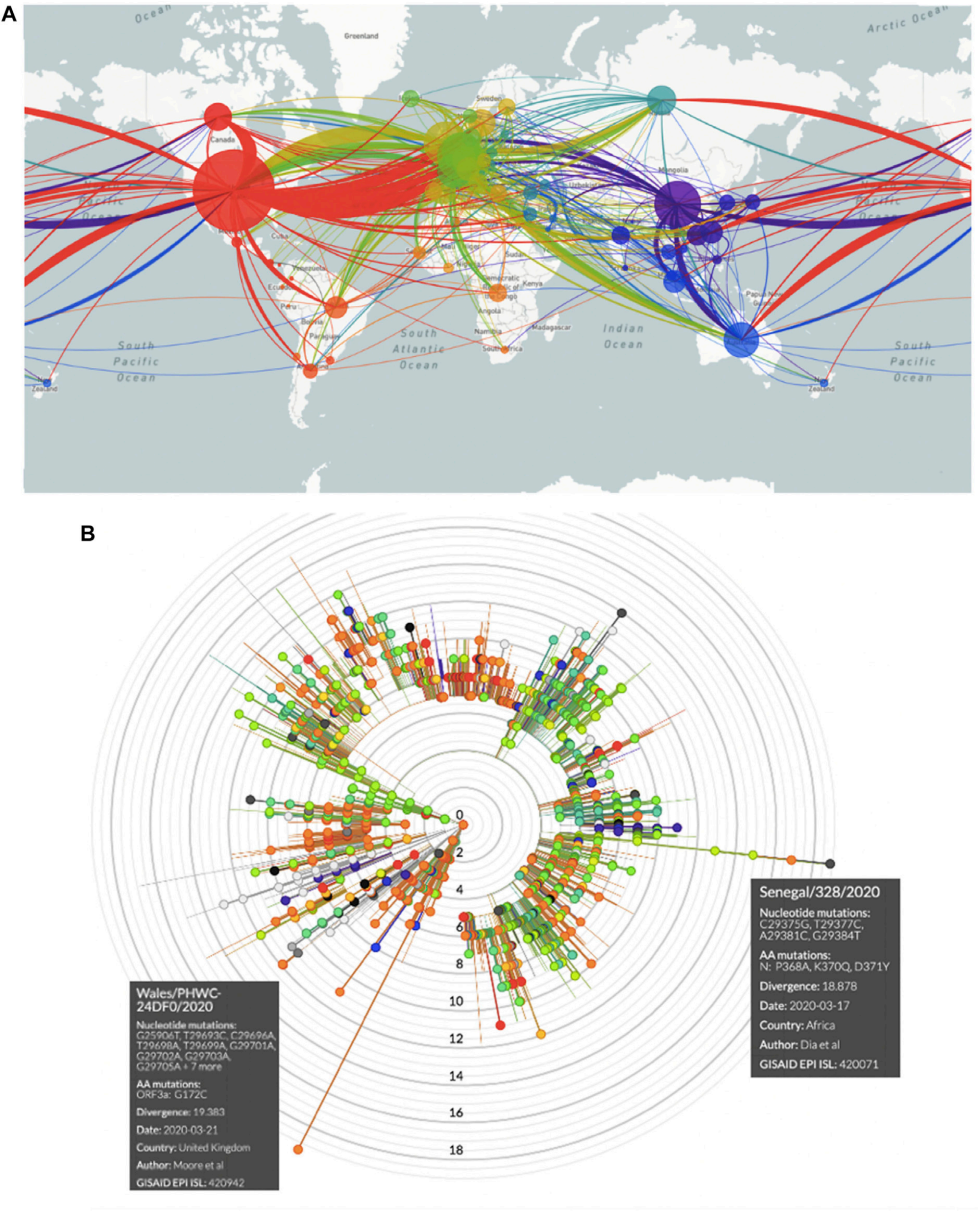
COVID-19 spreading and SARS-CoV-2 mutations. **(A)** Worldwide SARS-CoV-2 diffusion trajectories. Circle diameters are proportional to the number of virus isolates showing different sequences/acquired mutations. **(B)** Radial diagram of SARS-CoV-2 mutations worldwide. Concentric circles indicate the number of acquired genomic mutations detected in individual virus isolates. Each color identifies the geographical origin of the virus isolates.

Broadly diverse climate regions around the CET longitude (15°E) were severely exposed to COVID-19. Spain and Italy were the countries with the highest early incidence of COVID-19 in Europe (Figure 1, Supplementary Figure S1, Supplementary Table S3). The heaviest initial casualties in Italy were suffered by Lombardy and Veneto, i.e., cold and humid areas during wintertime. Markedly warmer and drier climate conditions prevail in Southern regions of the country. A further shift toward warmer/drier conditions occurs in Spain. Scandinavian countries provided a reference for cold winter temperatures, over a Sweden–Finland–Norway axis (Supplementary Table S4). This offered unique opportunities to assess a climate-dependent coronavirus infection model. Such analysis was performed at a whole-population level on 86,498 laboratory-confirmed SARS-CoV-2 infection cases in Italy, 64,095 in Spain, and 17,496 cases in Scandinavia (github.com/pcm-dpc/COVID-19) (Supplementary Appendix). Diffusion data in France (Supplementary Table S5), Germany (Supplementary Table S6), and the United Kingdom (Supplementary Table S7) provided a validation dataset, encompassing 210,239 COVID-19 cases. This model was then merged with that of coronavirus genetic evolution (Figure 2), for detecting signs of positive selection for increased aggressiveness across the analyzed regions. While such epidemiological models limit the analysis to available, correlated information, a whole-population analysis provided the largest possible data collection scale and was expected to average-out distinct demographic and social inhomogeneities.

## Methods

### Incidence Data

Data on laboratory-confirmed SARS-CoV-2 infection cases in Europe were collected at early time points of the pandemic/peak diffusion rates from the following sources: Italy (github.com/pcm-dpc/COVID-19), France (dashboard.covid19.data.gouv.fr/vue-d-ensemble?location=FRA), the United Kingdom (www.nhs.uk/), Germany (corona.rki.de), Spain (RTVE—Ministry of Health; www.rtve.es/noticias/20200415/mapa-del-coronavirus-espana/2004681.shtml), Sweden (Public Health Agency of Sweden; www.folkhalsomyndigheten.se/smittskydd-beredskap/utbrott/aktuella-utbrott/covid-19), Finland (National Institute for Health and Welfare THL; thl.fi/en/web/thlfi-en), and Norway (Norwegian Institute of Public Health; www.fhi.no/sv/smittsomme-sykdommer/corona/dags--og-ukerapporter/dags--og-ukerapporter-om-koronavirus).

SARS-CoV-2 virus spreading was modelled across a multitude of studies, (Baker et al., 2020; Hernandez-Vargas and Velasco-Hernandez, 2020; Kennedy et al., 2020; Kissler et al., 2020; Lipsitch et al., 2020; Pearson et al., 2020) (10.21203/rs.3.rs-82122/v1). However, essentially all current virus transmission models failed to predict regional heterogeneity. A recent article applied a susceptible-exposed-infectious-recovered (SEIR) compartmental mathematical model for predicting COVID-19 dynamics versus pathogen density in the environment and the use of preventive measures (Mwalili et al., 2020). The outcome of this modeling was the quantification of an R_0_ of 2.03, which implied that the pandemic would persist in the human population in the absence of control measures. However, while useful as a preventive model, its nature remained theoretical. The corresponding need for experimental validation by real-world observations applies to all virus diffusion models and to their provided risk estimates.

Our global, population-level study was designed to provide such evidence. Real-world data collection was utilized to quantify the impact of virus genetic drift versus environmental/regional determinants on COVID-19 diffusion. The main power of our analysis was its population-wide nature, using data-analysis procedures designed to tame the impact of the main confounding factors. Our approach had limits, as demographic, socioeconomic, and behavioral details were not available in list mode-as individual-associated variables and could only be tackled indirectly.

The key grounds for our strategy were as follows:

I. An explosive diffusion of SARS-CoV-2 in Western Europe occurred early along the course of the pandemic, providing a vast number of infection cases, over parallel calendar timeframes.
II. High-quality disease-reporting procedures allowed whole-population-level analyses, with the inclusion of 378,328 laboratory-confirmed SARS-CoV-2 infection cases across continental Europe and the United Kingdom.
III. Our large-number, whole-population analysis was adopted to tame the impact of demographic and social inhomogeneities. The county/province level of analysis was correspondingly adopted to reveal systematic territorial inhomogeneities if the above had not been the case.
IV. The North–South span of the European regions involved in the early phases of COVID-19 provided a vast array of climatic zones. The null hypothesis was challenged that COVID-19 transmission velocity would have been the same across climate areas, quantitatively categorized as an independent variable.
V. As viral evolution processes in specific geographic areas (Eletreby et al., 2020) can effectively mimic climate region-associated spreading (Li et al., 2020b; Korber et al., 2020; Rausch et al., 2020; Volz et al., 2021), these processes were analysed accordingly.
VI. A potent confounding factor in disease transmission analyses is the founder effect, i.e., the date of the first moving of an infectious case to a geographic site. Thus, all collected data were normalized versus the date of the first detection of infection cases in each region analysed. This effectively prevented bias associated with founder effects and with traveling modes and intensity.
VII. Normalization of infection cases to the first detection date allowed us to assess doubling rates of COVID-19 cases in each analysed area, independently from the absolute size of the population analysed (Carleton et al., 2021).
VIII. Large-scale virus diffusion containment measures were expected to be a main confounding factor. Hence, we directed our search toward the initial period of explosive diffusion of the virus and ended our observations at the time of the first modulation of infectious case incidence rates, upon implementation of containment measures. This was implemented in a region-by-region manner (Carleton et al., 2021).

Disease severity was then classified as 1) hospitalized cases, 2) intensive care unit patients, 3) recovered cases, and 4) deaths. These findings were presented as cumulative incidence by region.

The cumulative incidence of COVID-19 cases was then linked to Köppen–Geiger climate classification maps (koeppen-geiger.vu-wien.ac.at/present.htm). These were computed as a mean parametrization of data collected between 1980 and 2016 (Beck et al., 2018). The tripartite classification by country areas was compounded as an independent variable versus COVID-19 doubling time (Table 1).

**TABLE 1:** COVID-19 doubling time versus climate area.

Country/region	COVID-19 doubling time (days)	Climate area	Lab-confirmed case numbers ^*^
Spain	4.2	Csa/Csb/Bsk	64,095
Southern Italy	5.38	Csa/Csb	5,322
Central Italy	5.87	Csa/Cfa/Cfb	10,842
Northern Italy	6.63	Cfa/Cfb	70,334
Germany	7.0	Cfb	73,522
France	7.5	Cfb	68,665
United Kingdom	7.2	Cfb	68,052
Sweden	9.4	Dfc/Cfb	11,321
Finland	10.8	Dfc/Dfb	2,646
Norway	12.95	Dfc/Dfb/ET	5,855

* According to the Köppen–Geiger climate classification maps. Csa: Mediterranean hot summer climate; Csb: Mediterranean warm/cool summer climates; Bsk: cold semi-arid climate; Cfa: humid subtropical climate; Cfb: oceanic climate; Dfc: subarctic or boreal climates; Dfb: warm summer continental or hemiboreal climates; ET: Tundra climate.

** Laboratory-confirmed SARS-CoV-2 infection cases in Europe cases were retrieved by country at peak diffusion rates, before the landmark dates indicated: Italy (github.com/pcm-dpc/COVID-19, March 27, 2020), France (dashboard.covid19.data.gouv.fr/vue-d-ensemble?location=FRA; April 4, 2020), the United Kingdom (www.nhs.uk/; April 9, 2020), Germany (corona.rki.de; April 2, 2020), Spain (RTVE—Ministry of Health; www.rtve.es/noticias/20200415/mapa-del-coronavirus-espana/2004681.shtml; March 27, 2020), Sweden (Public Health Agency of Sweden; www.folkhalsomyndigheten.se/smittskydd-beredskap/utbrott/aktuella-utbrott/covid-19; April 9, 2020), Finland (National Institute for Health and Welfare THL; thl.fi/en/web/thlfi-en; April 7, 2020), and Norway (www.fhi.no/sv/smittsomme-sykdommer/corona/dags--og-ukerapporter/dags--og-ukerapporter-om-koronavirus; April 14, 2020).

### SARS-CoV-2 Mutation Analysis

SARS-CoV-2 genomic RNA sequences and country-correlated data were obtained from nextstrain.org/ncov/global. Each data point was represented as a bead, whereby each bead corresponded to a specific set of virus mutations (mutation haplotype) (Figure 2, Supplementary Figures S2–S9). “Beads-on-a-string” plots were then generated, which represented a linked series of individual mutation haplotypes that acquired subsequent mutations over time. Phylogeny trees for such mutation clusters were then obtained for drawing distinct evolutionary branches of SARS-CoV-2 (nextstrain.org/ncov/europe?branchLabel=aa) (Figure 2, Supplementary Figures S2–S9).

### Statistical Analysis

The cumulative incidence (Cimoli et al., 2004; Ambrogi et al., 2006) of COVID-19 cases versus calendar dates was acquired at the province/county level. Corresponding plots acted as a smoother for accurate determination of infection curve parameters. They also served to average urban versus countryside population dynamics/events on a province-by-province basis. Disease cumulative incidence graphs were found to largely follow a peculiar linear growth pattern (Thurner et al., 2020). This allowed to rigorously apply linear regression methodology for determining case-incidence rates.

At subsequent time points, deviations from linearity, with flattening of disease incidence curves, were recorded, following implementation of country-wide restrictions in traveling and social interactions (www.gazzettaufficiale.it/eli/gu/2020/03/08/59/sg/pdf). These inflection points were taken as landmark dates and marked the end of the observation period. From each one of these dates, the doubling time for the cumulative number of diagnoses was calculated backward for each province, as follows. Two dates were identified: the maximum date, at which the cumulative number of diagnoses were lower than a half of the cumulative number of diagnoses at the landmark time, and the minimum date, with a cumulative number of diagnoses greater than half of the cumulative number of diagnoses at the landmark date. The fraction of days from the minimum date to achieve half of the cumulative number of diagnoses at the landmark date was obtained by a linear assumption for the cumulative incidence between the two dates. Correspondingly, distinct calendar dates were applied to data collection in different provinces, regions, and countries, according to the spreading sequence of the pandemic. Of note, each of these estimates corresponded to the fastest spreading velocity of COVID-19 in each region.

Coefficients, standard error, and 95% CIs were computed. Percentile distribution boxplots of COVID-19 case doubling times were drawn. Median, maximum value, minimum value, and distribution outliers were estimated. The correlation between COVID-19 spreading rates versus normalized climate-area values was computed by ANOVA.

### Software

Stata software version 16 was used for data importing, manipulation, and graphics (StataCorp, 2019, *Stata Statistical Software: Release 16*, College Station, TX: StataCorp LLC).

## Results

### COVID-19 Case Doubling Time by Geographic Area

Infection transmission rates were computed for the following:

Italy: on COVID-19 cases from March 3 to March 27, 2020 (n = 86,498) (Supplementary Appendix) (Supplementary Figures S10–S12)
Spain: on COVID-19 cases from February 25 to March 27, 2020 (n = 64,095) (Supplementary Figure S13)
Norway: on data (>50 cumulative infection case outbreaks) obtained from February 21 to April 14, 2020 (n = 6,676) (Supplementary Figure S14)
Finland: on COVID-19 cases from March 1 to April 7, 2020 (n = 2,646) (Supplementary Figure S15)
Sweden: on data (>50 cumulative infection case outbreaks) obtained from February 26 to April 9, 2020 (n = 8,995) (Supplementary Figure S16)
France: on COVID-19 cases from February 25 to April 4, 2020 (Supplementary Figure S17)
United Kingdom: on COVID-19 cases from February 1 to April 9, 2020 (Supplementary Figure S17)
Germany: on COVID-19 cases from February 24 to April 2, 2020 (Supplementary Figure S17)

### COVID-19 Doubling Time Versus Climate Region

Quantitative climate assessments are affected by complex, interdependent sets of variables (Babin, 2020). Up to 89 distinct parameters are required for meteorological classification alone (apps.ecmwf.int/datasets/data/interim-full-moda/levtype=sfc/). Discrete humidity measures, temperature profiles (papers.ssrn.com/sol3/papers.cfm?abstract_id=3556998) (Sundell et al., 2016; Sajadi et al., 2020), and weather structure intertwine with lifestyle, social, and occupational determinants (Sajadi et al., 2020) (www.medrxiv.org/content/10.1101/2020.03.23.20040501v4). Hence, fundamental sources of uncertainty are associated with climate modeling (Baker et al., 2020). We thus resorted to utilizing the Köppen–Geiger climate classification (koeppen-geiger.vu-wien.ac.at/present.htm), as drawn over 30+ years of observations and as robustly validated in the literature (Beck et al., 2018; Babin, 2020; Briz-Redón and Serrano-Aroca, 2020; Chakrabarti et al., 2020). The Köppen–Geiger climate classification was summarized as a tripartite classification by country/region/province, which was compounded as an independent variable versus COVID-19 spreading velocity (Table 1).

**FIGURE 3:**
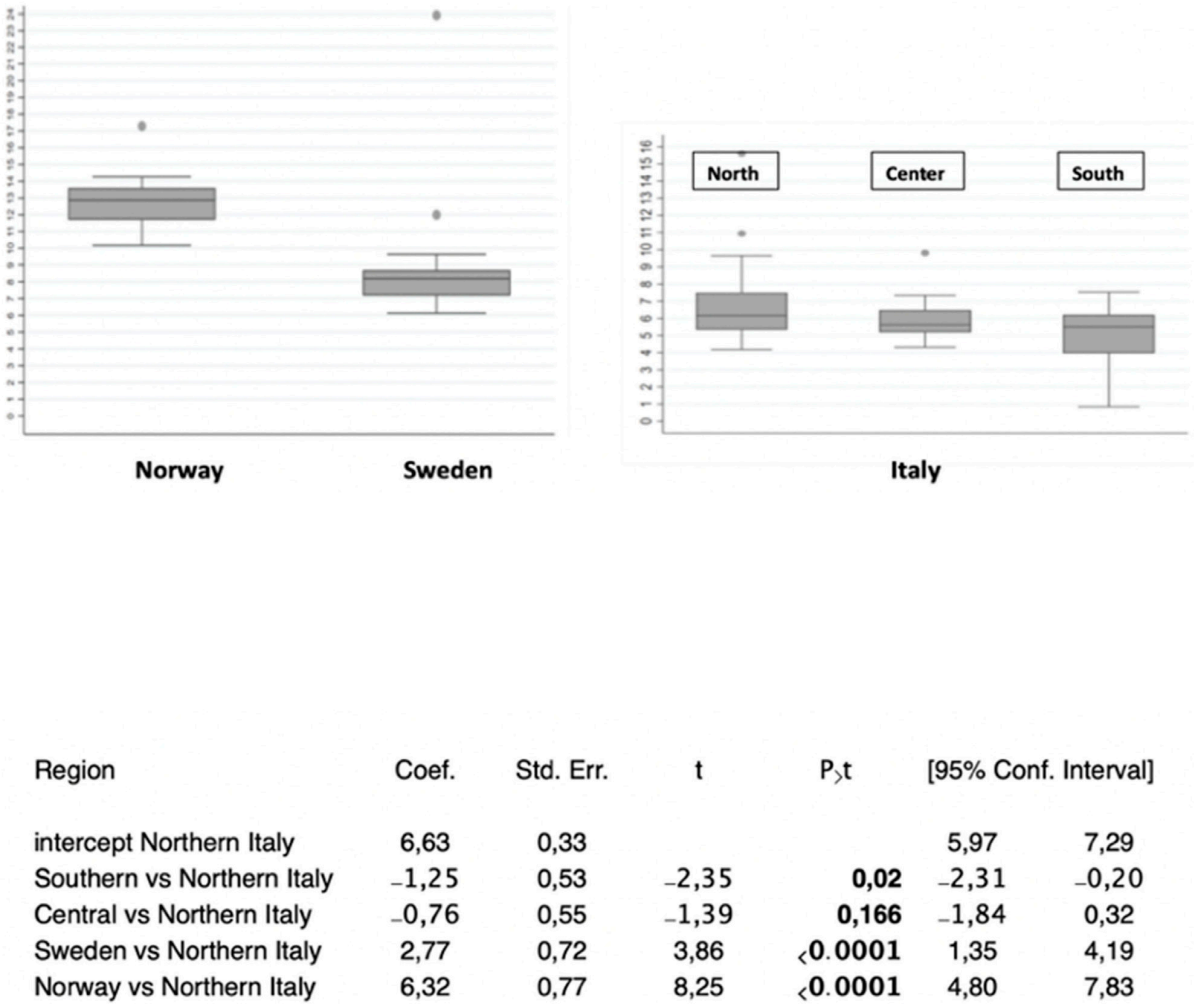
COVID-19 diffusion across geographic areas. **(top)** Distribution boxplots of doubling times of COVID-19 cases in the areas/countries analyzed. **(bottom)** Doubling times of COVID-19 cases versus the Northern Italy benchmark, which corresponds to the central intercept. Coef.: coefficient; Std. Err.: standard error; 95% confidence intervals are shown. P>t: P value of comparison versus the benchmark.

Cumulative numbers of COVID-19 cases versus calendar dates were normalized to the highest case incidence in each area (Supplementary Figures S10–S17). Pandemic doubling times were correspondingly computed (Supplementary Table S3) (Carleton et al., 2021) and grouped by geographic region. The average doubling time for Northern Italy was 6.63 (SD = 1.94) days, 5.87 (SD = 1.08) days in Central regions, and 5.38 (SD = 2.31) days in Southern areas, for significantly shorter doubling time in Southern regions (*p* = 0.02 versus Northern Italy) (Supplementary Table S3, Figure 3, Supplementary Figures S10–S12). The mean COVID-19 doubling-time for the whole country was 6.06 (SD = 1.95) days.

**FIGURE 4:**
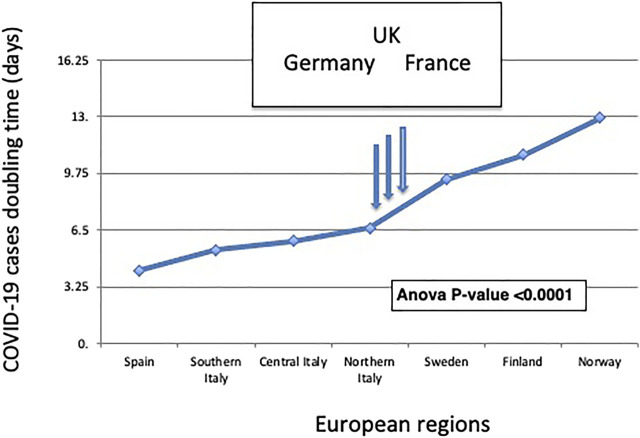
The North-South gradient of COVID-19. The doubling time of COVID-19 cases according to distinct geographic areas is shown. European regions are listed from left to right according to their classification by climate zone (Table 1). The ANOVA *p*-value for the association of the plotted values to climate zones is shown. The vertical arrows indicate the COVID-19 doubling times in the validation datasets of (from left to right) Germany, the United Kingdom, and France.

With a doubling time of 4.2 days, Spain extended such a trend (Supplementary Figure S13). At the opposite end of the climate spectrum, Scandinavia showed longer COVID-19 doubling times, over a Sweden–Finland–Norway axis, with a doubling time of 9.4 days (SD = 1.2) for Sweden (*p* < 0.0001 versus Northern Italy), 10.8 days for Finland, and 12.95 days (SD = 0.52) for Norway (*p* < 0.0001 versus Northern Italy) (Figure 3, Supplementary Figures S14–S16). This depicted a distinct North–South gradient of COVID-19 spreading velocity (ANOVA *p* < 0.0001) (Figure 4; Table 1).

This climate model was challenged versus a validation dataset of 210,239 laboratory-confirmed COVID-19 cases in Germany, France, and the United Kingdom, whose average climate areas fall in between classification classes of Northern Italy and Southern Sweden. Germany, France, and the United Kingdom provided a potentially highly diverse set of reference regions. This notwithstanding, the pandemic doubling time was computed to be 7.0 days in Germany (Supplementary Figure S17), 7.5 days in France, and 7.2 days in the United Kingdom. This fell in between Northern Italy and Sweden data, in full consistency with the predictions of our model.

Disease severity as classified hospitalization, intensive care unit, and fatality rates were compounded as cumulative incidence by region (Supplementary Figure S12). However, analysis of neither disease onset severity nor outcome provided correlation with parameters of regional diffusion heterogeneity. It should be noted that data on recoveries and deaths are not consistently classified across all the regions under study and are considered less reliable than those of confirmed COVID-19 cases (Carleton et al., 2021).

### SARS-CoV-2 Genetic Drift-Driven Diffusion

Sequence mutation analysis revealed different branches of acquired mutations, i.e., distinct groups of viral genome mutations (haplotypes), at sites of major diffusion in Europe (nextstrain.org/ncov/europe) (Figure 2, Supplementary Figures S2–S9). Each of these branches was observed to acquire additional mutations over time, in an uneven manner among different geographic areas. We searched this dataset for potential indicators of positive selection for specific virus mutation(s). One virus mutation, i.e., the spike D614G amino acid change, was associated with increased COVID-19 aggressiveness (Korber et al., 2020). The variant D614G was first found in samples collected on January 3, 2020, in the United States. However, strains with the mutation were found in many parts of the world, at approximately the same time, suggesting that the mutation already existed in China and then spread across the world. The D614G mutation appeared early in Europe (inferred date, January 6, 2020) (nextstrain.org/ncov/; www.gisaid.org/) (Korber et al., 2020) and was subsequently found to spread evenly across European countries.

Among descendants of D614G viruses, we looked for evidence of positive selection, by investigating the potential impact of additional mutations. It should be noted that most viral mutations may not have phenotypic effects, as most of them are probably neutral or near neutral. Further, while some mutations may become dominant over time, the overall diversity of SARS-CoV-2 genomes will continue to increase due to genetic drift. Nevertheless, if positive selection for one or more virus mutations had been at work, deviation from even distributions of virus descendants across regions had to be expected. Among flags of such unevenness, we looked for 1) mutation-correlated increase of disease severity over time, 2) prevalence of such mutation(s) in the hardest-hit countries, and 3) progressively broader diffusion of more aggressive virus genotypes along the early course of the pandemic.

The highest numbers of accumulated mutations were revealed in SARS-CoV-2 in Wales and Senegal isolates (Figure 2B). Hence, they most likely represented late correlates of viral genetic drift over time. Of interest, the lowest number of accumulated mutations was recorded in Italy, a country with high disease severity in Europe. This appeared poorly consistent with a progressive increase of disease severity upon accumulation of novel mutations, suggesting instead correlation with an initially short SARS-CoV-2 evolution time. Large mutation loads were observed in Spain (n = 14), the second hardest-hit country in Europe. However, a similar mutation load was observed in Sweden (n = 13), a country with much more limited COVID-19 transmission and severity, further supporting a correlation with genetic drift. Consistent, large mutation loads were observed in late-disease-insurgence countries, such as France and Belgium (n = 16), supporting a slow SARS-CoV-2 genomic evolution, along the course of the disease (Supplementary Figures S3–S9). Complex patterns were subsequently detected at later time points of SARS-CoV-2 diffusion.

## Discussion

Large efforts have gone into modelling COVID-19 transmission, according to global and local population dynamics, demographics, governmental policies, and infectious ability of the virus (Lipsitch et al., 2020; Pearson et al., 2020; Hernandez-Vargas and Velasco-Hernandez, 2020; Kennedy et al., 2020; Baker et al., 2020; Kissler et al., 2020; Rausch et al., 2020; Volz et al., 2021, #34796; Korber et al., 2020; Li et al., 2020b; Mwalili et al., 2020). Most models, though, showed an inadequate capacity for predicting the regional/climate-associated diffusion dynamics of the pandemic (Baker et al., 2020).

We speculated that fundamental variables associated with the COVID-19 uneven diffusion remained to be identified and set a search for discovering such factor(s). We went on to perform a population-wide analysis, on 378,328 laboratory-confirmed SARS-CoV-2 infection cases in continental Europe and the United Kingdom. A robust determination required collecting epidemiological data (Cimoli et al., 2004; Ambrogi et al., 2006), before intervention via disease-containment measures. We thus went on to identify landmark dates, as inflection points of disease incidence curves associated with disease-taming procedures, throughout Western Europe. This led us to identify a quasi-universal pattern of linear growth of COVID-19 cases over time in most of the regions analysed. Such a growth pattern was not predicted by most pandemic spreading models (Carleton et al., 2021), supporting the analytical validity of our novel data collection strategy. This also suggested a unique diffusion mode of SARS-CoV-2 (Thurner et al., 2020), which was largely robust to conventional modeling of disease diffusion dynamics. Within such a unique diffusion mode of SARS-CoV-2, distinct COVID-19 transmission rates were identified as associated with different geographic regions.

Still, the accumulation of mutations of SARS-CoV-2 may have led to distinct selection for disease progression over different regions. An indicator of selective pressure for viral evolution has been that of progressively larger prevalence across different geographic locations (Korber et al., 2020), as indicated for the spike D614G mutation. However, D614G was associated with higher upper respiratory tract viral loads, but a much more limited impact was found in early analyses on disease severity (Li et al., 2020b; Korber et al., 2020; Volz et al., 2021). This appeared puzzling (Rausch et al., 2020), as higher viral loads have been associated with worse disease courses (Yu et al., 2020) and with increased mortality (Westblade et al., 2020). The hypothesis of positive selection of spike D614G was further investigated in the United Kingdom using more than 25,000 whole-genome SARS-CoV-2 sequences. This indicated 614G increases in frequency relative to 614D as consistent with a selective advantage, but not in all cases (Volz et al., 2021). This suggested that “a combination of evolutionary selection for G614 and the founder’s effects of being introduced into highly mobile populations may have together contributed in part to its rise” (Korber et al., 2020). These findings were recapitulated “as a slow genetic drift of a highly stable [SARS-CoV-2] genome” (Dearlove et al., 2020; Rausch et al., 2020), during the early timeframes of the pandemic.

We looked for further evidence of selection for viral evolution, utilizing broader indicators of the predominance of specific mutation(s) versus disease severity. Four major mutation groups/haplotypes were revealed in all examined European countries. The highest number of accumulated mutations was revealed in Wales and Senegal SARS-CoV-2 isolates, suggesting a correlation with genetic drift, at late stages of the disease. The lowest number of accumulated mutations was recorded in Italy, the country that first showed severe disease outbreaks in Europe, Furthermore, similar mutation loads were observed in Spain, the second hardest-hit country in Europe, and Sweden, a country with much less explosive COVID-19 transmission, suggesting a correlation with disease duration, rather than with selection for higher disease severity. The larger mutation loads were revealed in France and Belgium, both late-disease-insurgence countries, further supporting a relationship between mutation acquisition and length of the disease course. Taken together, our findings add evidence to a model of SARS-CoV-2 genetic drifting during the early course of the pandemic.

COVID-19 spreading models based on population demographics and socioeconomic factors all systematically failed to account for regional diffusion heterogeneity during the pandemic. Our findings show a sharp North–South gradient, with the shortest COVID-19 doubling times in Southern Italy and Spain. At the opposite end of the climate spectrum, Scandinavia showed the longest COVID-19 doubling times, over a Sweden–Finland–Norway axis. This climate model was verified in country-wide validation datasets of COVID-19 cases in Germany, France, and the United Kingdom, which included 210,239 laboratory-confirmed SARS-CoV-2 infection cases. This showed pandemic doubling times that were intermediate between Northern and Southern regions and that were in sharp consistency with the climate-area Köppen–Geiger model (Beck et al., 2018). Thus, our findings support a climate dependency of COVID-19 transmission capacity, usefully adding to the set of variables that are involved in modulating SARS-CoV-2 diffusion.

Findings of a more efficient coronavirus spreading in warmer regions are consistent with the resilience of coronaviruses to high-temperature environmental conditions (Chin et al., 2020) [Kampf, 2020 #33396van Doremalen, 2020 #33393]. Of note, the Middle East Respiratory Syndrome (MERS) was first reported in Saudi Arabia (www.cdc.gov/coronavirus/mers). MERS is caused by the MERS-CoV, which is structurally and genetically related to SARS-CoV, indicating that at least some coronavirus strains may better propagate in high-temperature climate conditions (www.cdc.gov/coronavirus/mers/risk.html).

However, climate areas are associated with complex sets of variables, among them indoor versus outdoor temperature profiles, specific/relative/absolute humidity (Sundell et al., 2016; Babin, 2020; Sajadi et al., 2020), UV exposure versus daily time/season/latitude (Carleton et al., 2021; Sajadi et al., 2020), weather structure, and ventilation, together with social behavior, inter-individual distancing, indoor crowding, lifestyle, and outside physical activity (papers.ssrn.com/sol3/papers.cfm?abstract_id=3556998) (Sajadi et al., 2020) versus community structure, socioeconomic and healthcare factors (www.medrxiv.org/content/10.1101/2020.03.23.20040501v4), including traveling or traveling modes. Recent studies have begun to dissect such determinants, indicating the impact of UV exposure on COVID-19 transmission (Carleton et al., 2021), while suggesting no role of temperature and humidity (SSRN 3567840, 2020; papers.ssrn.com) (Briz-Redón and Serrano-Aroca, 2020; Carleton et al., 2021). Our research confirms these findings and the lack of impact of outside temperature on COVID-19 progression over the areas analyzed (unpublished observations). Additional work is expected to bring in further insight into climate area-associated virus diffusion determinants.

Taken together, our findings suggest higher SARS-CoV-2 resilience in warmer regions than previously predicted and caution that high environmental temperatures may not efficiently tame SARS-CoV-2 infectiousness (Kissler et al., 2020). Very cold regions may be better spared by recurrent courses of COVID-19.

## Data Availability

The original contributions presented in the study are included in the article/Supplementary Material. Further inquiries can be directed to the corresponding author.
