# Supplementary material for: Genetic Drift Versus Climate Region Spreading Dynamics of COVID-19
Source: Front Genet. 2021 Dec 23;12:663371. doi: 10.3389/fgene.2021.663371 (PMC8740632; doi:10.3389/fgene.2021.663371)
Supplement: Supplementary file 1 [file DataSheet1.pdf]

Supplemental Appendix

Genetic drift versus climate region spreading dynamics of COVID-19

R. Di Pietro [1], M. Basile [1], L. Antolini [2] and S. Alberti [3]

[1] Department of Medicine and Aging Sciences, Section of Biomorphology, G. d'Annunzio University of Chieti-Pescara, Italy (r.dipietro@unich.it) (mariangelabasile@hotmail.it).
[2] Center for Biostatistics, Department of Clinical Medicine, Prevention and Biotechnology, University of Milano-Bicocca, Monza, Italy (laura.antolini@unimib.it).
[3] Unit of Medical Genetics, Department of Biomedical Sciences - BIOMORF, University of Messina, via Consolare Valeria, Messina, Italy.

Drs. Di Pietro and Basile contributed equally to this article.

Supplemental Figures

Figure S1. Worldwide temperature distributions and climate zones.

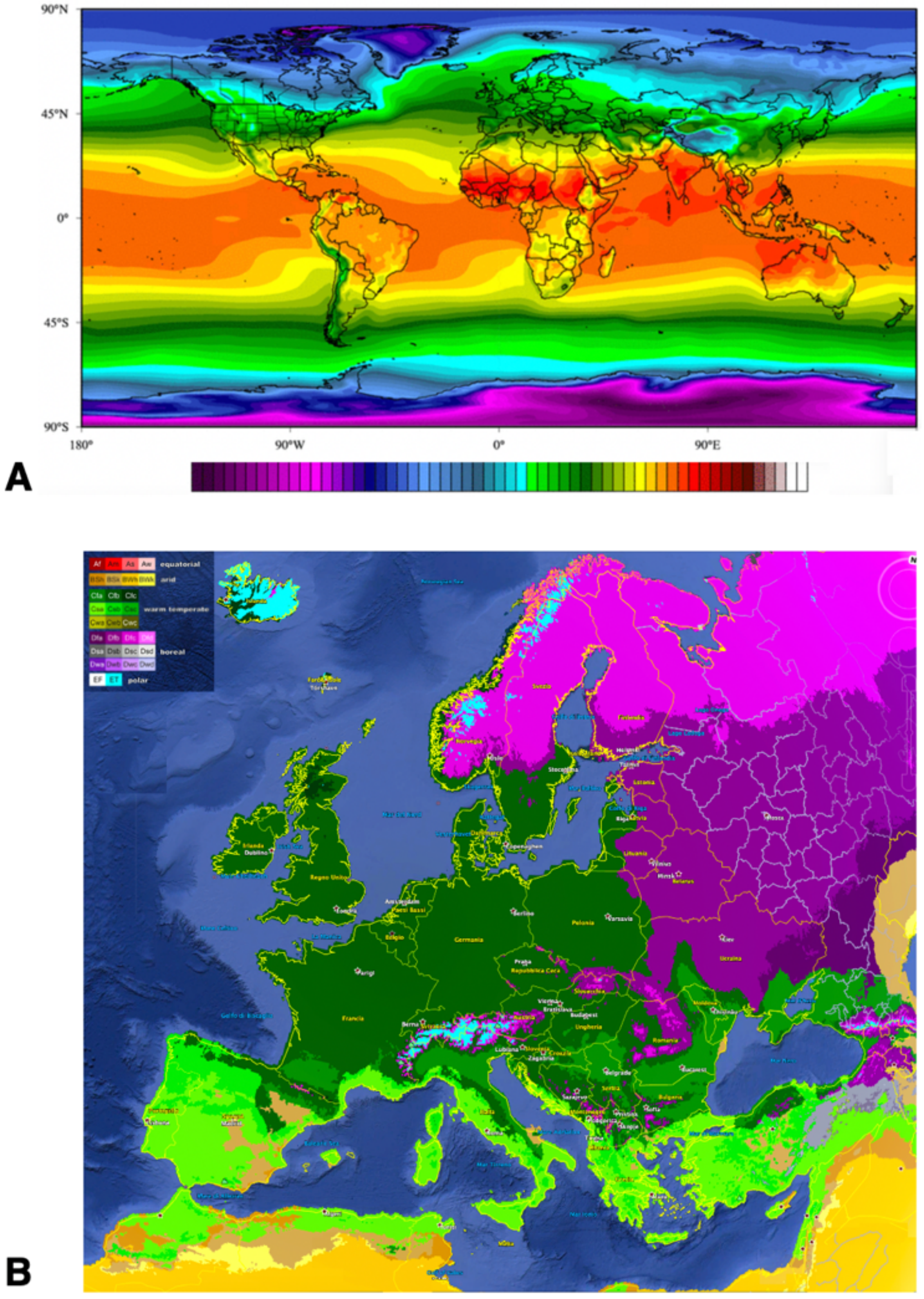

(**A**) Worldwide temperature averages versus geographic areas are indicated in pseudocolors. Average temperatures are indicated for early spring. Temperature color codes are indicated below the graph. (**B**) Köppen–Geiger climate classification map of Europe. (**inset**) Climate areas color codes.

Figure S2. Global accumulation of genomic mutations of SARS-CoV-2.

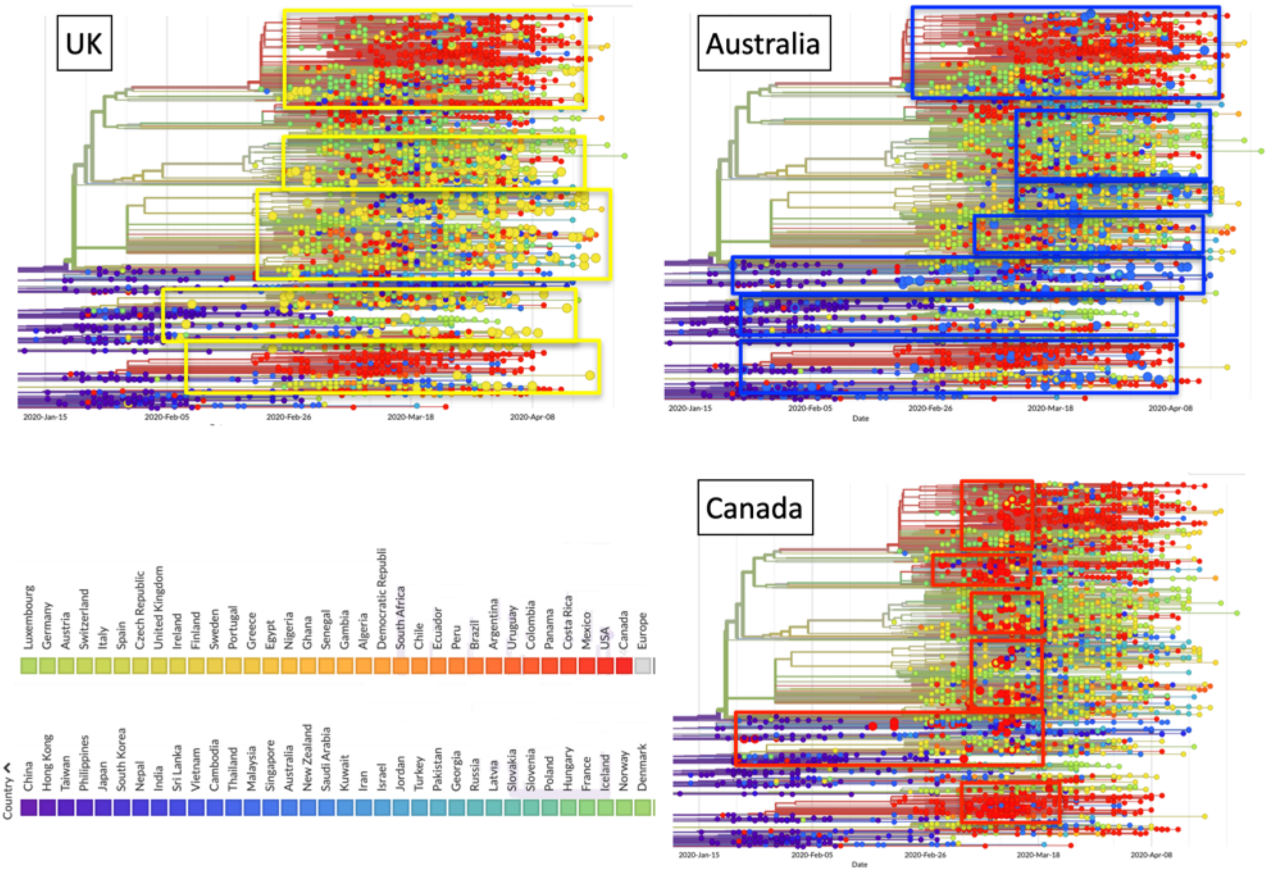

Accumulation of genomic mutations of SARS-CoV-2 over time around the globe, at the early time points of the pandemic. Each data-point is represented as a bead, whereby each bead corresponds to a specific set of virus mutations (mutation haplotype) (nextstrain.org/ncov/).

(**bottom left**) Country color codes. Beads and bead sequences were color-coded, according to the country where the virus sample was isolated from. The 'beads-on-a-string' plots link successions of viral mutations, i.e. mutation haplotypes that acquired additional mutations over time. Phylogeny trees for such mutations are presented, that draw distinct evolutionary branches of SARS-CoV-2 over calendar dates, which are indicated at the bottom of each graph. Larger beads indicate mutations identified in the indicated country. UK: bright yellow; Australia: blue; Canada: red. Rectangles enclose mutations evolutionary branches by country.

Figure S3. Genomic mutations of SARS-CoV-2 in Europe.

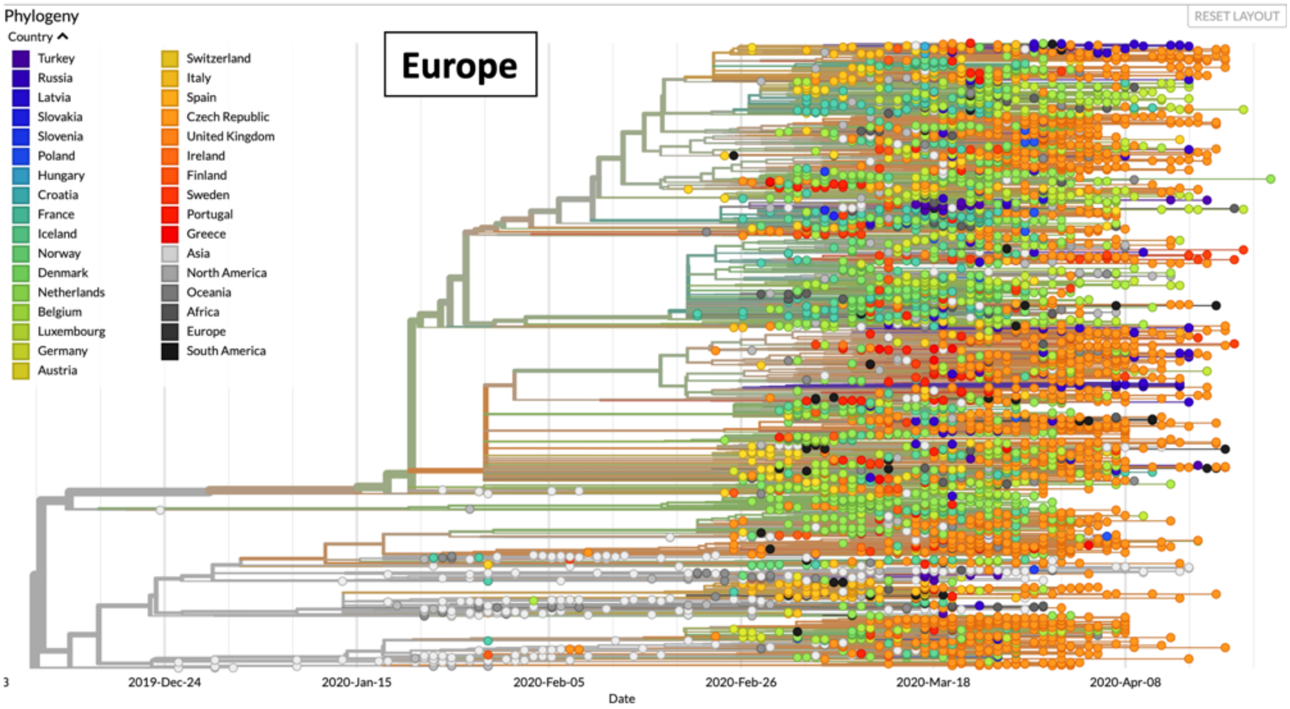

Accumulation of genomic mutations of SARS-CoV-2 over time in Europe, at the early time points of the pandemic. Each data-point is represented as a bead, whereby each bead corresponds to a specific set of virus mutations (mutation haplotype). Beads and bead sequences are color-coded, according to the country where the virus sample was isolated from (upper left). The 'beads-on-a-string' plots link successions of viral mutations, i.e. mutation haplotypes that acquired additional mutations over time. Phylogeny trees for such mutations are presented, that draw distinct evolutionary branches of SARS-CoV-2 over calendar dates, which are indicated at the bottom of each graph (nextstrain.org/ncov/europe?branchLabel=aa).

Figure S4. Genomic mutations of SARS-CoV-2 - Spain.

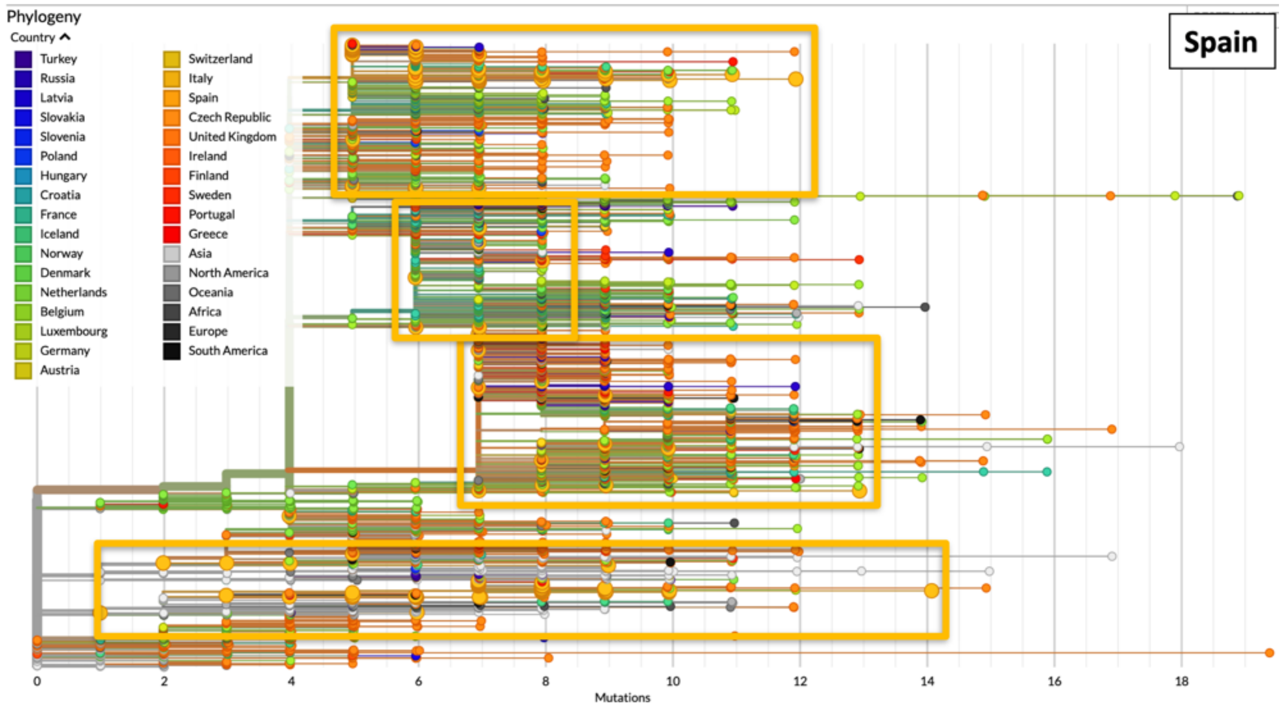

The accumulation of genomic mutations of SARS-CoV-2 over time in Spain, at the early time points of the pandemic, is shown as strings of dark-yellow large dots. Each data-point is represented as a bead, whereby each bead corresponds to a specific set of virus mutations (mutation haplotype). Beads and bead sequences are color-coded, according to the country where the virus sample was isolated from (upper left). The 'beads-on-a-string' plots link successions of viral mutations, i.e. mutation haplotypes that acquired additional mutations over time. Phylogeny trees for such mutations are presented, that draw distinct evolutionary branches of SARS-CoV-2 (nextstrain.org/ncov/europe?branchLabel=aa). Dark-yellow rectangles enclose successions of viral mutations in Spain in each phylogenetic branch.

The number of accumulated mutations in individual viral isolates is indicated at the bottom.

Figure S5. Genomic mutations of SARS-CoV-2 - Italy.

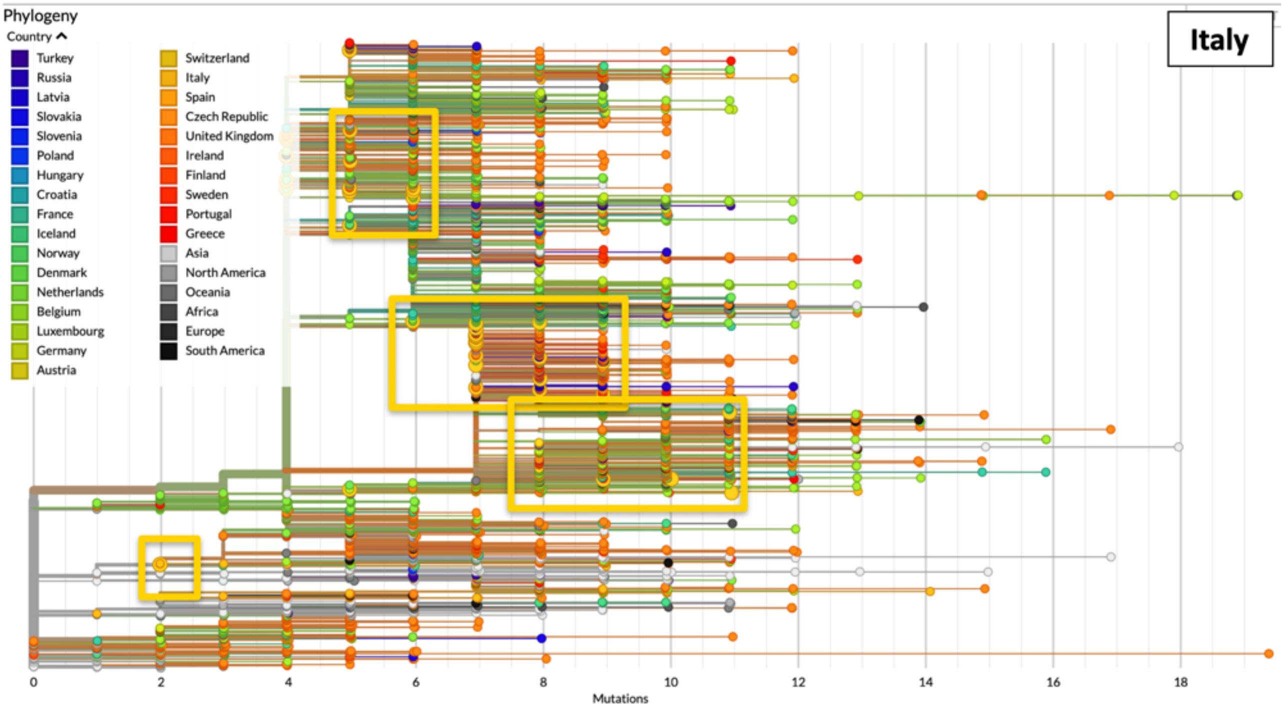

The accumulation of genomic mutations of SARS-CoV-2 over time in Italy, at the early time points of the pandemic, is shown as strings of yellow large dots. Each data-point is represented as a bead, whereby each bead corresponds to a specific set of virus mutations (mutation haplotype). Beads and bead sequences are color-coded, according to the country where the virus sample was isolated from (upper left). The 'beads-on-a-string' plots link successions of viral mutations, i.e. mutation haplotypes that acquired additional mutations over time. Phylogeny trees for such mutations are presented, that draw distinct evolutionary branches of SARS-CoV-2 (nextstrain.org/ncov/europe?branchLabel=aa). Yellow rectangles enclose successions of viral mutations in Italy in each phylogenetic branch. The number of accumulated mutations in individual viral isolates is indicated at the bottom.

Figure S6. Genomic mutations of SARS-CoV-2 - Sweden.

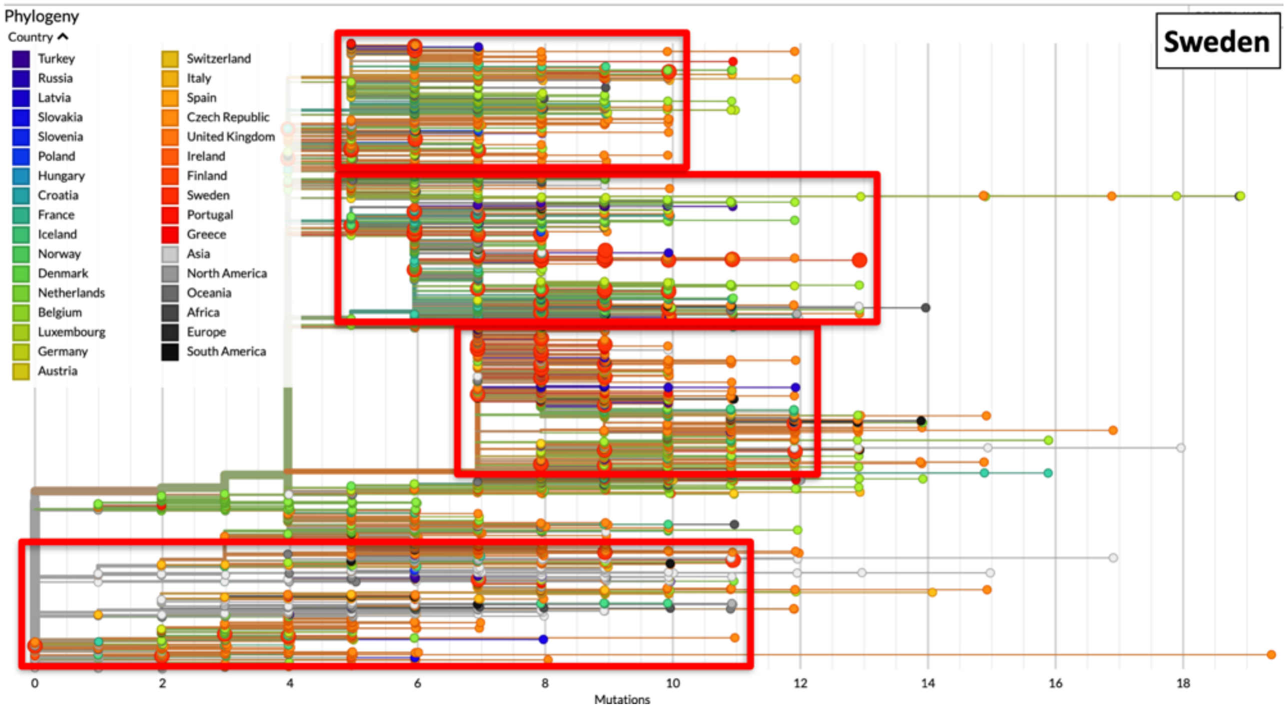

The accumulation of genomic mutations of SARS-CoV-2 over time in Sweden, at the early time points of the pandemic, is shown as strings of red large dots. Each data-point is represented as a bead, whereby each bead corresponds to a specific set of virus mutations (mutation haplotype). Beads and bead sequences are color-coded, according to the country where the virus sample was isolated from (upper left). The 'beads-on-a-string' plots link successions of viral mutations, i.e. mutation haplotypes that acquired additional mutations over time. Phylogeny trees for such mutations are presented, that draw distinct evolutionary branches of SARS-CoV-2 (nextstrain.org/ncov/europe?branchLabel=aa). Red rectangles enclose successions of viral mutations in Sweden in each phylogenetic branch.

The number of accumulated mutations in individual viral isolates is indicated at the bottom.

Figure S7. Genomic mutations of SARS-CoV-2 - The Netherlands.

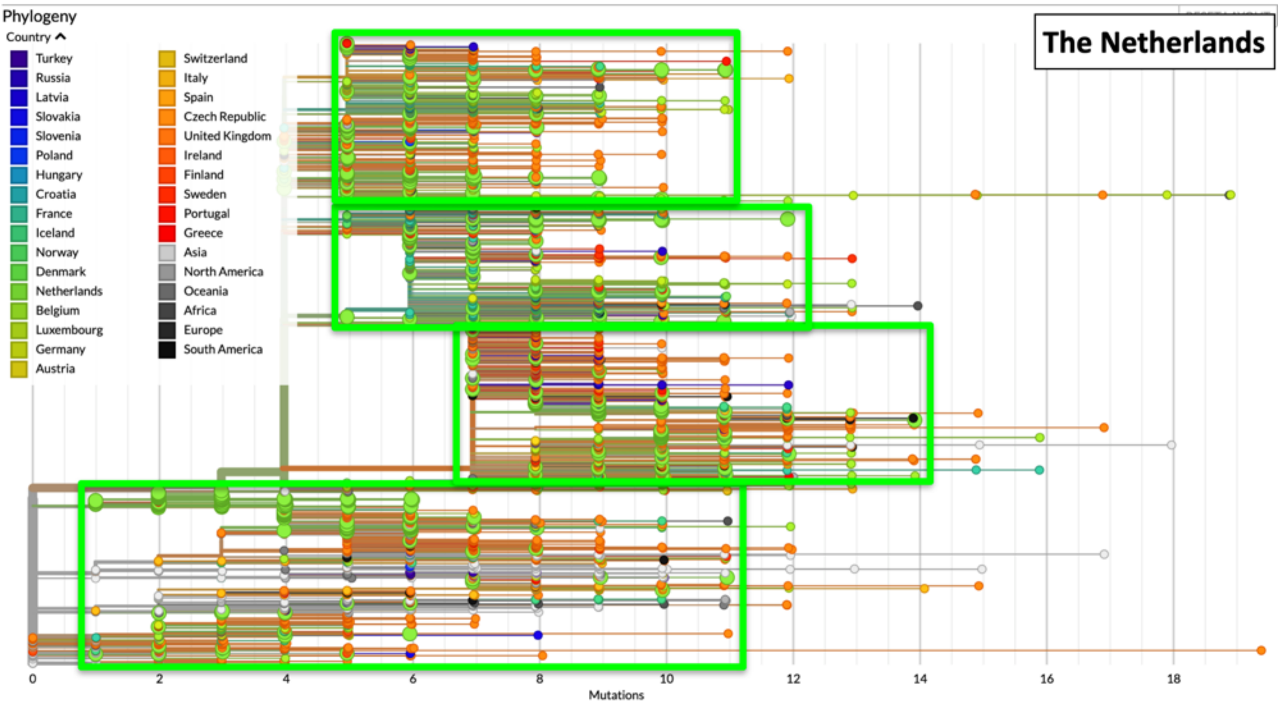

The accumulation of genomic mutations of SARS-CoV-2 over time in The Netherlands, at the early time points of the pandemic, is shown as strings of bright-green large dots. Each data-point is represented as a bead, whereby each bead corresponds to a specific set of virus mutations (mutation haplotype). Beads and bead sequences are color-coded, according to the country where the virus sample was isolated from (upper left). The 'beads-on-a-string' plots link successions of viral mutations, i.e. mutation haplotypes that acquired additional mutations over time. Phylogeny trees for such mutations are presented, that draw distinct evolutionary branches of SARS-CoV-2 (nextstrain.org/ncov/europe?branchLabel=aa). Bright-green rectangles enclose successions of viral mutations in The Netherlands in each phylogenetic branch.

The number of accumulated mutations in individual viral isolates is indicated at the bottom.

Figure S8. Genomic mutations of SARS-CoV-2 - Belgium.

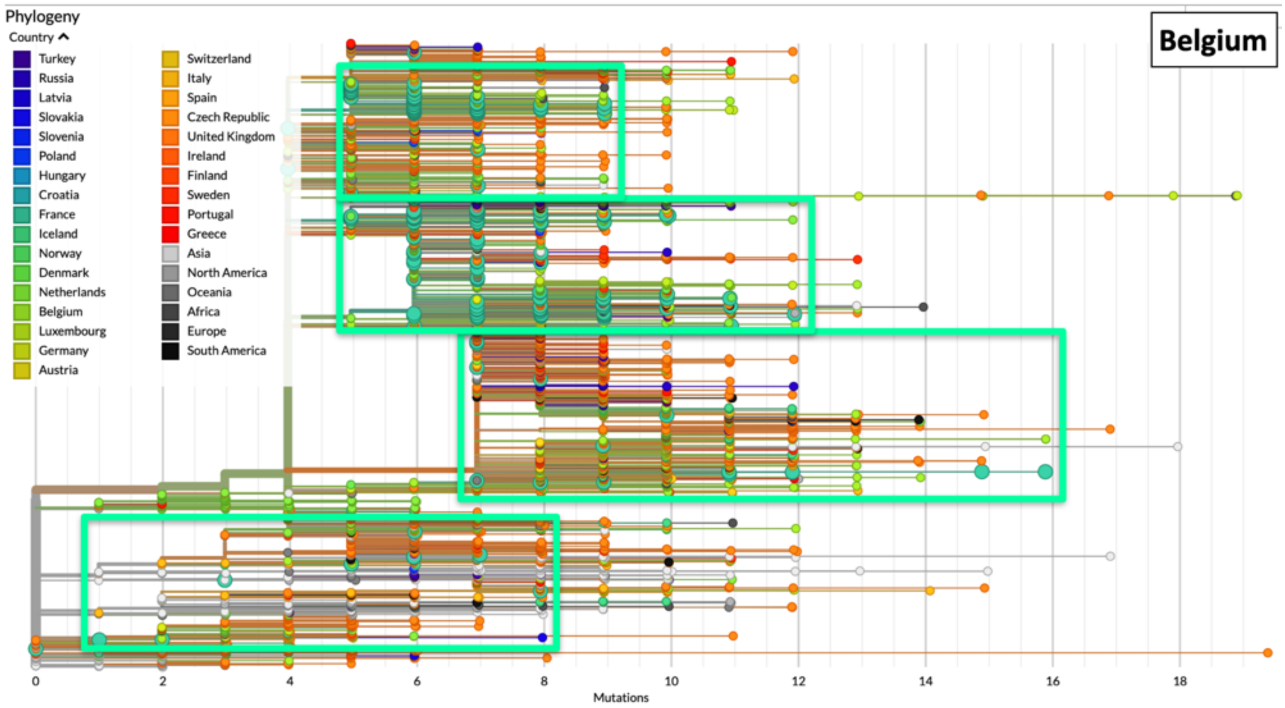

The accumulation of genomic mutations of SARS-CoV-2 over time in Belgium, at the early time points of the pandemic, is shown as strings of light-green large dots. Each data-point is represented as a bead, whereby each bead corresponds to a specific set of virus mutations (mutation haplotype). Beads and bead sequences are color-coded, according to the country where the virus sample was isolated from (upper left). The 'beads-on-a-string' plots link successions of viral mutations, i.e. mutation haplotypes that acquired additional mutations over time. Phylogeny trees for such mutations are presented, that draw distinct evolutionary branches of SARS-CoV-2 (nextstrain.org/ncov/europe?branchLabel=aa). Light-green rectangles enclose successions of viral mutations in Belgium in each phylogenetic branch.

The number of accumulated mutations in individual viral isolates is indicated at the bottom.

Figure S9. Genomic mutations of SARS-CoV-2 - France.

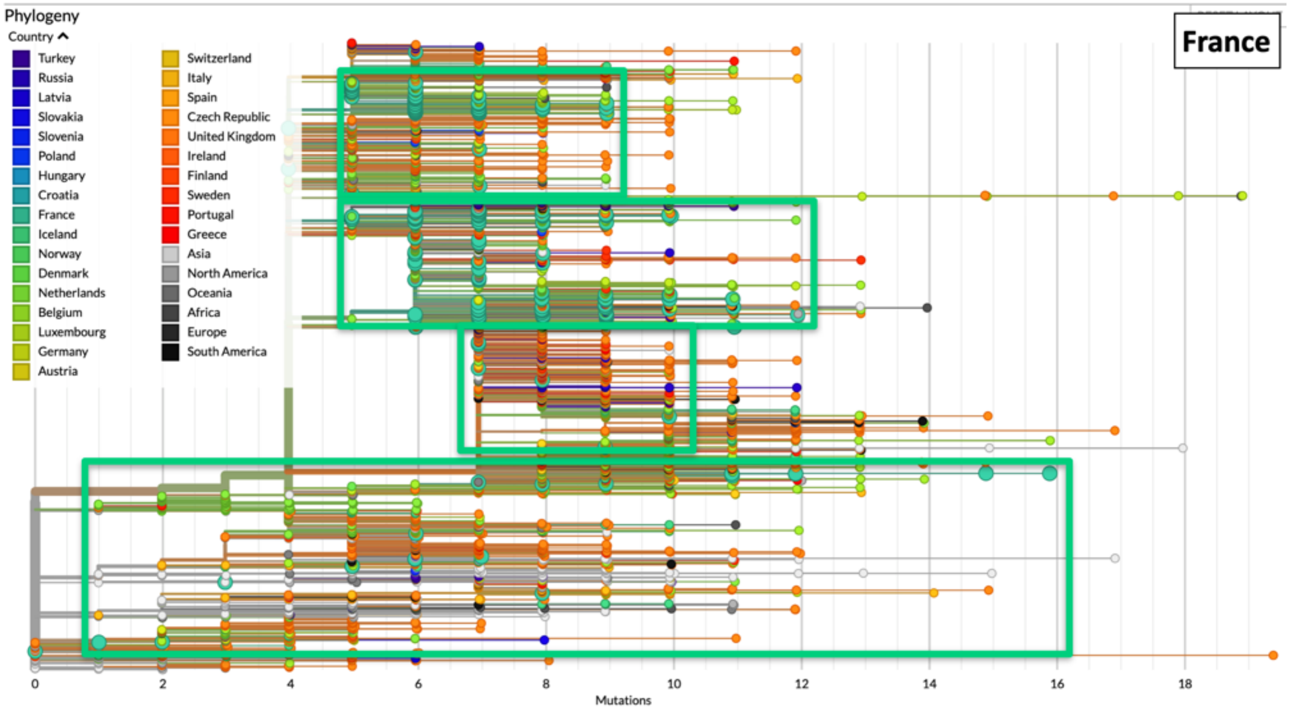

The accumulation of genomic mutations of SARS-CoV-2 over time in France, at the early time points of the pandemic, is shown as strings of deep-green large dots. Each data-point is represented as a bead, whereby each bead corresponds to a specific set of virus mutations (mutation haplotype). Beads and bead sequences are color-coded, according to the country where the virus sample was isolated from (upper left). The 'beads-on-a-string' plots link successions of viral mutations, i.e. mutation haplotypes that acquired additional mutations over time. Phylogeny trees for such mutations are presented, that draw distinct evolutionary branches of SARS-CoV-2 (nextstrain.org/ncov/europe?branchLabel=aa). Deep-green rectangles enclose successions of viral mutations in France in each phylogenetic branch.

The number of accumulated mutations in individual viral isolates is indicated at the bottom.

Figure S10. Progression of COVID-19 over Italy - Northern Provinces ***.**

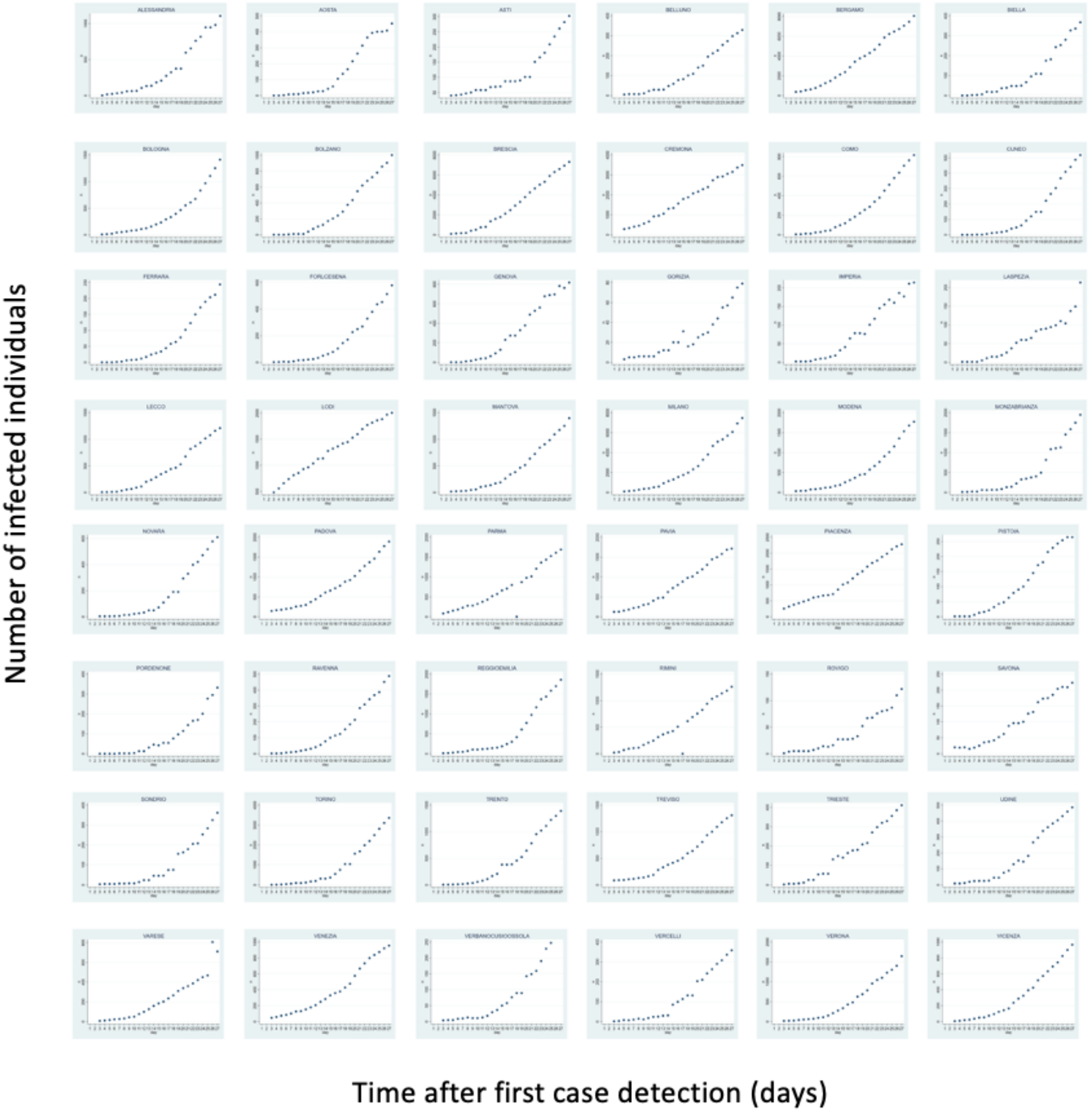

*: Cumulative case incidence in the indicated municipalities is plotted versus time (days, from March 3rd to March 27th, 2020).
For comparison purposes, all graphs were normalized versus the highest number of cases per province. The doubling times of COVID-19 cases were computed on non-normalized, absolute numbers of infection cases.
Graphs are in alphabetical order by province name.

Figure S11. Progression of COVID-19 over Italy - Central & Southern Provinces***.**

Central provinces

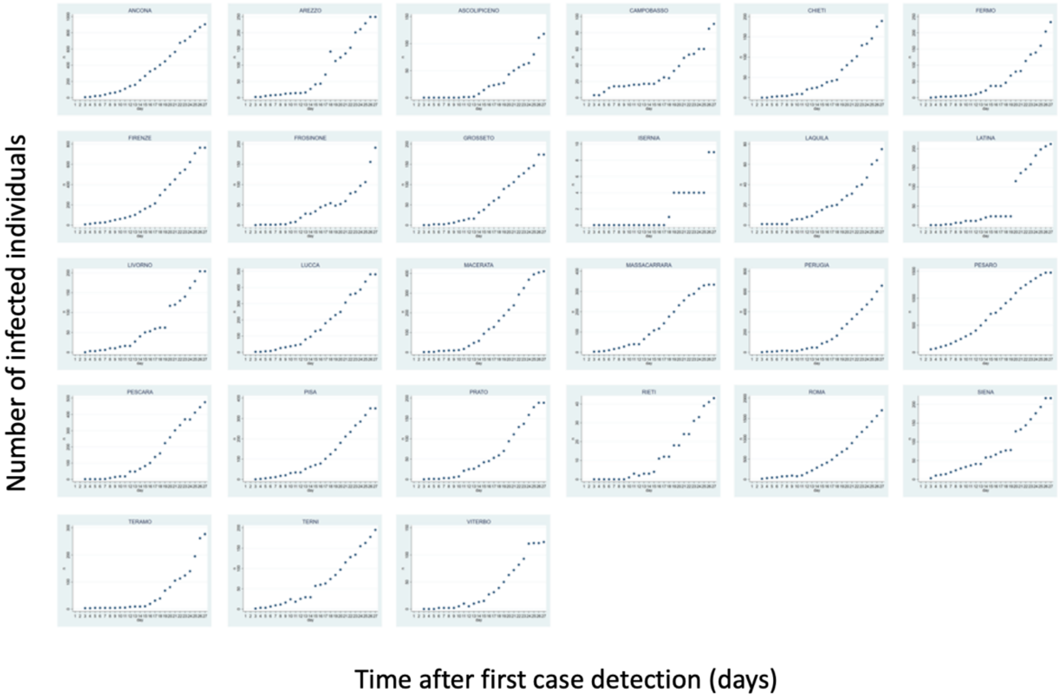

Southern provinces

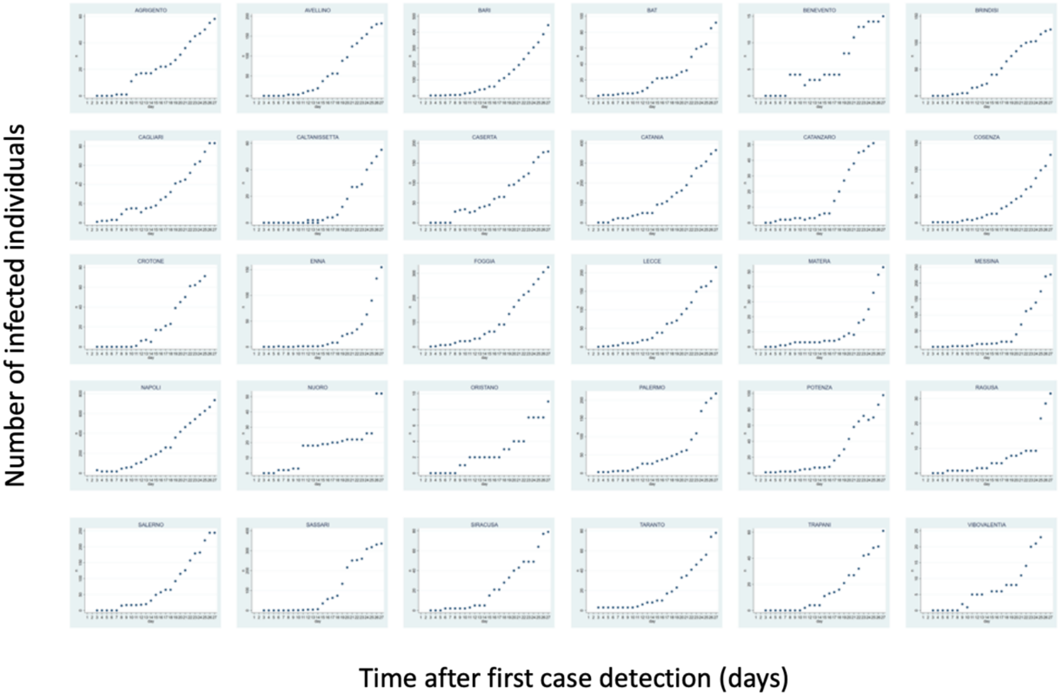

*: Cumulative case incidence in the indicated municipalities is plotted versus time (days, from March 3rd to March 27th, 2020). For comparison purposes, all graphs were normalized versus the highest number of cases per Province. The doubling times of COVID-19 cases were computed on non-normalized, absolute numbers of infection cases. Graphs are in alphabetical order by province name.

Figure S12. Progression of COVID-19 throughout Italy – global data by region*.

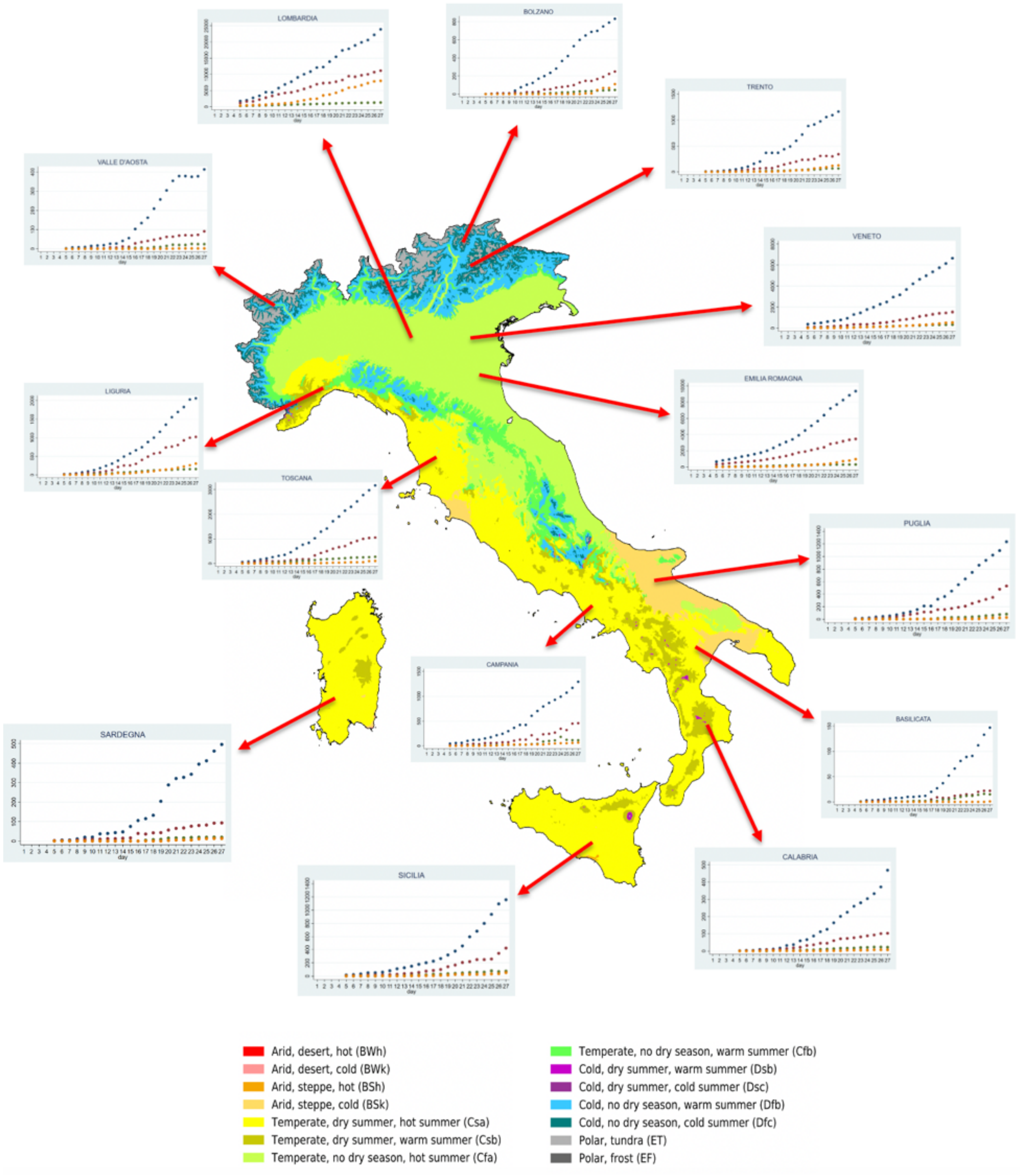

Köppen–Geiger climate classification map of Italy. (**red arrows**) Data on COVID-19 spreading and disease outcome in individual regions (days; calendar time from March 3rd to March 27th, 2020) are overlaid over the country's climate areas. (**insets**) Dark gray dots: SARS-CoV-2-positive cases; brown dots: hospitalized cases; green dots: intensive-care unit cases; orange dots: recovered cases. (**bottom**) Color codes for climate areas classification.

Figure S13. Progression of COVID-19 in Spain*.

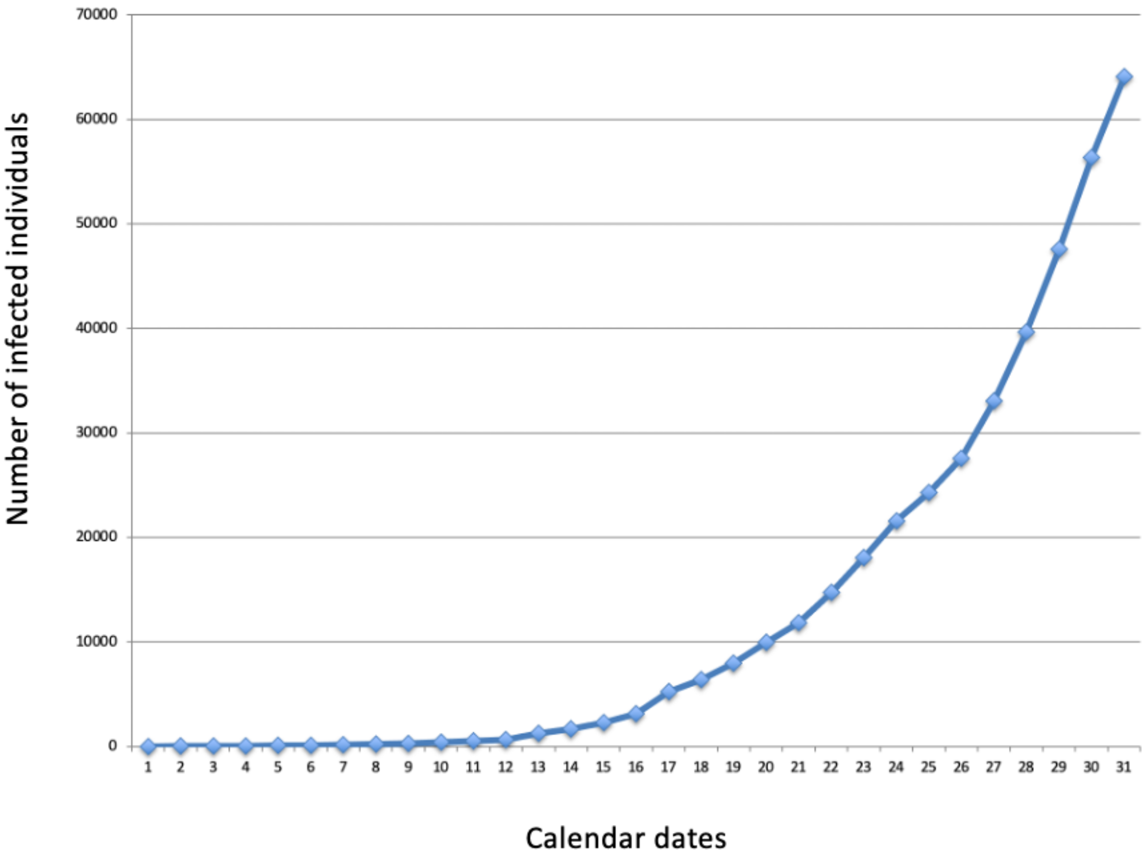

*: Cumulative case incidence in Spain is plotted versus calendar dates (March 1-31, 2020).

Figure S14. Progression of COVID-19 in Norway*.

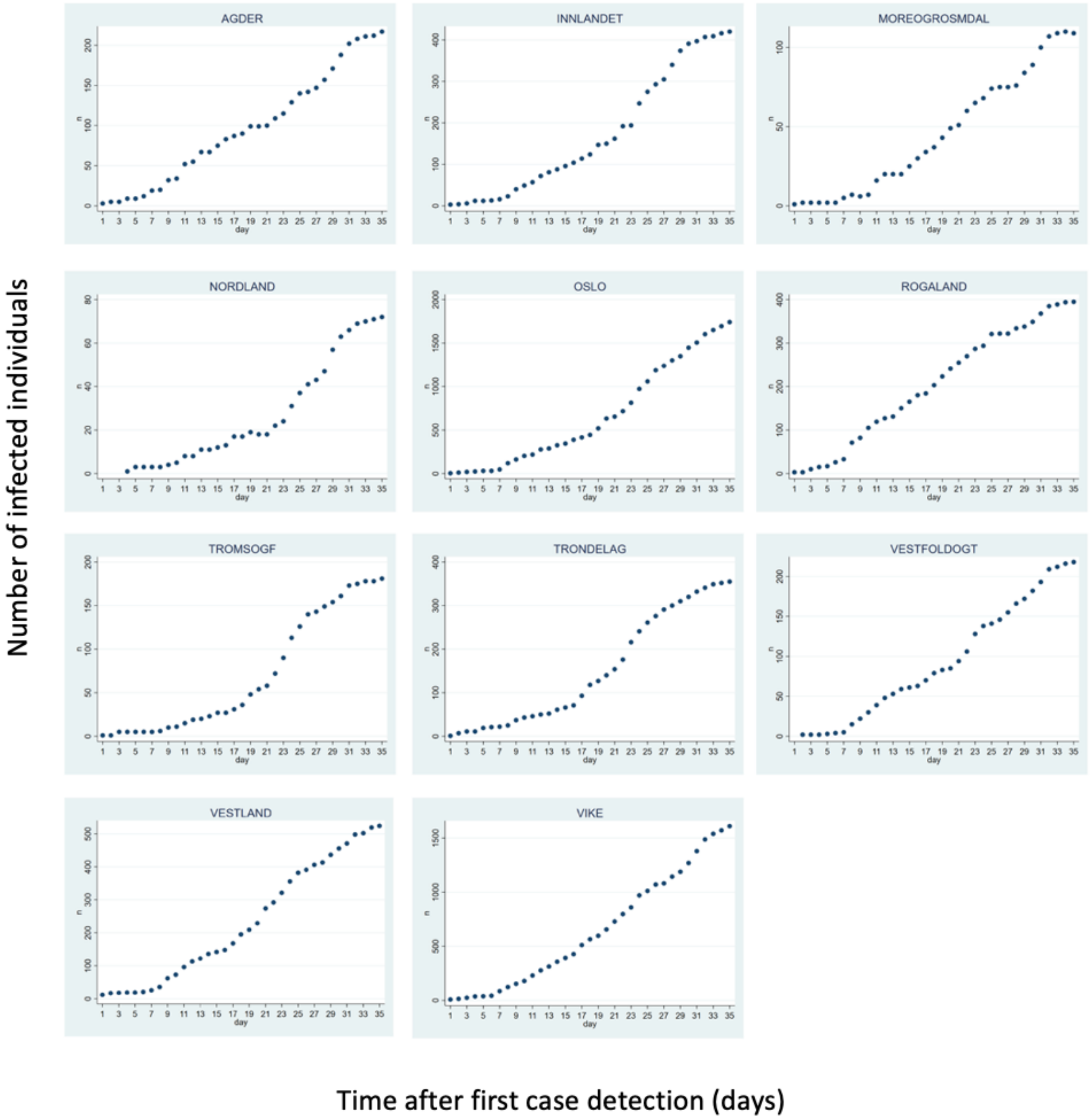

*: Cumulative case incidence in the indicated municipalities is plotted versus time (days; calendar dates from March 1st to April 7th, 2020). For comparison purposes, all graphs were normalized versus the highest number of cases per province. The doubling times of COVID-19 cases were computed on non-normalized, absolute numbers of infection cases. Graphs are in alphabetical order by province name.

Figure S15. Progression of COVID-19 in Finland*.

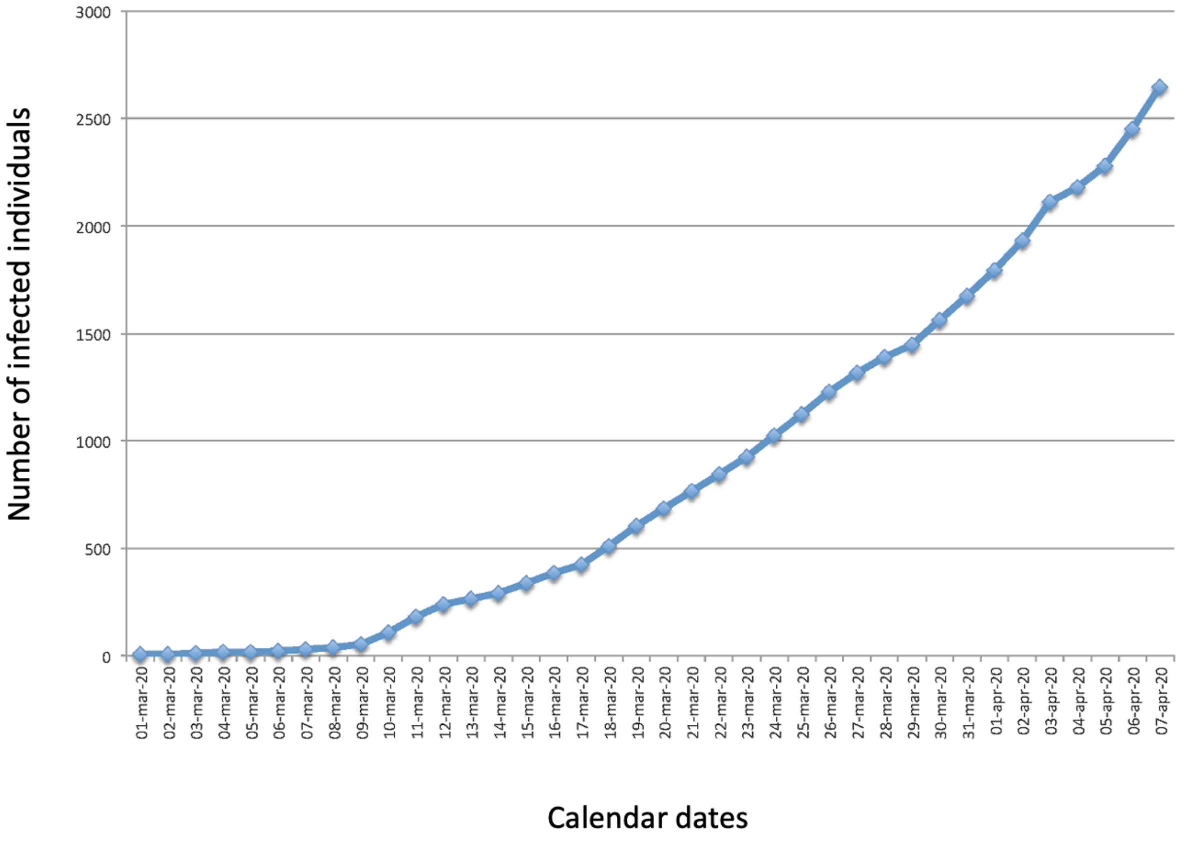

*: Cumulative case incidence in the indicated municipalities is plotted versus calendar dates (March 1-April 7, 2020).

Figure S16. Progression of COVID-19 over Sweden*.

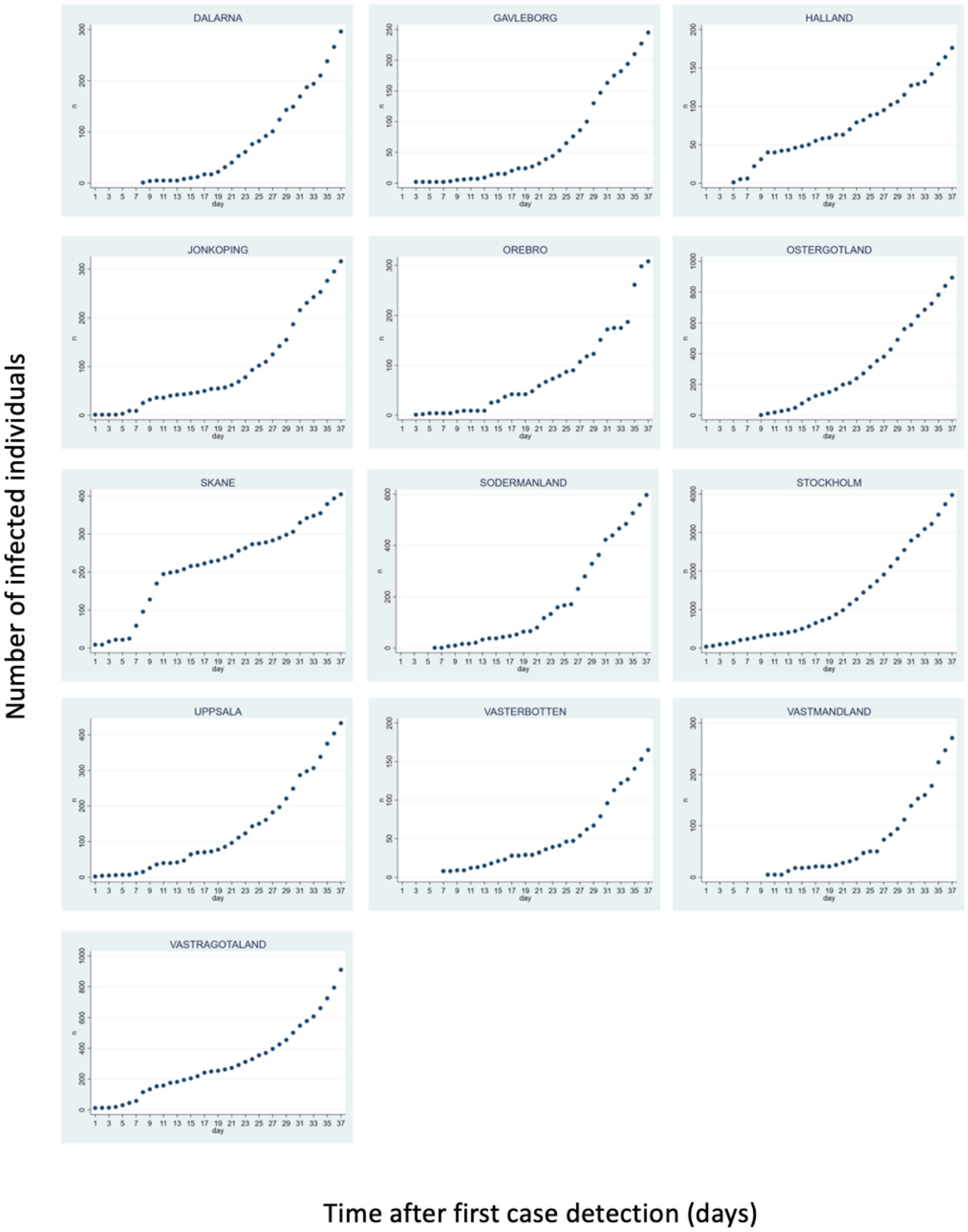

*: Cumulative case incidence in the indicated municipalities is plotted versus time (days; calendar dates from March 1st to March 23rd, 2020). For comparison purposes, all graphs were normalized versus the highest number of cases per province. The doubling times of COVID-19 cases were computed on non-normalized, absolute numbers of infection cases. Graphs are in alphabetical order by province name.

Figure S17. Progression of COVID-19 in France, Germany and UK*.

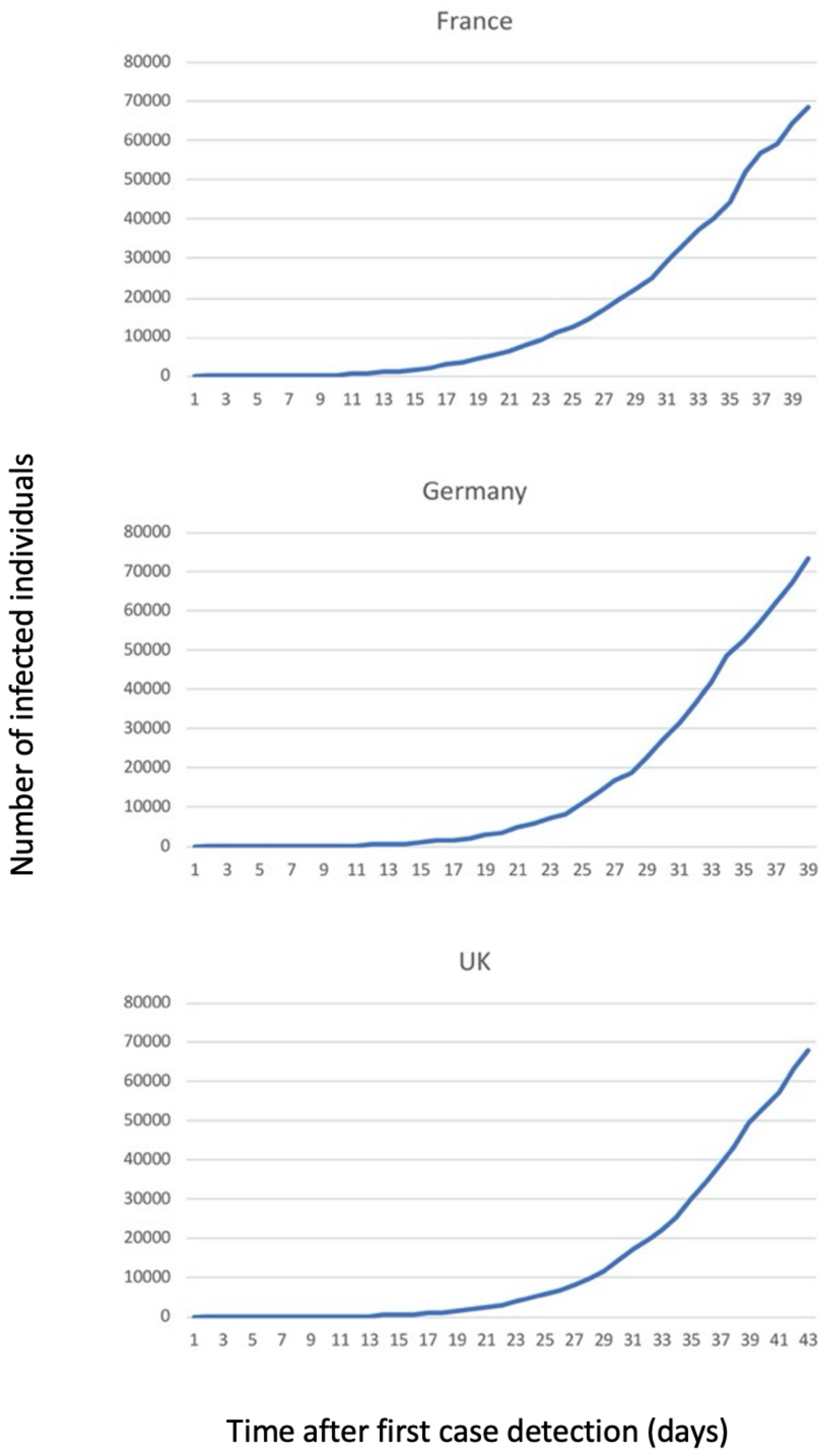

*: Laboratory-confirmed infection cases in Europe cases were assessed country by country before landmark dates (last data-point in each graph): France (dashboard.covid19.data.gouv.fr/vue-d-ensemble?locatio n=FRA; April 4th 2020); Germany (corona.rki.de; April 2nd 2020); UK (www.nhs.uk/; April 9th 2020). Cumulative case incidence is plotted over time (days).

Supplemental online Table 1. COVID-19 spreading in Central and South America.

	Argentina	Brazil	Chile	Colombia	Costa Rica	Dominican Republic	Ecuador	Mexico	Panama	Peru	Uruguay	Venezuela	Trinidad and Tobago
March 1*		2				1	1	2					
March 2		2				1	1	5					
March 3		2	1			1	6	5					
March 4	1	2	1			1	7	5					
March 5	1	3	1		1	1	7	5					
March 6	1	7	1	1	1	1	16	5					
March 7	2	13	5	1	1	1	14	5		1			
March 8	9	19	5	1	5	1	14	7		6			
March 9	12	25	10	1	9	1	15	7		6			
March 10	12	25	13	3	9	5	15	7	1	9			
March 11	17	34	17	3	13	5	15	7	8	11			
March 12	19	52	23	9	13	5	17	11	10	17			
March 13	31	77	33	9	22	5	17	12	14	22			
March 14	34	98	43	16	23	5	23	26	27	28		2	1
March 15	45	121	61	24	23	5	23	41	27	43		2	1
March 16	56	200	75	24	23	5	37	53	43	71	4	2	2
March 17	65	234	156	45	41	21	58	53	69	86	6	33	5
March 18	65	234	156	45	41	21	58	82	69	86	6	33	5
March 19	79	291	238	93	50	21	155	93	86	145	29	36	7
March 20	97	428	342	108	87	34	199	118	109	234	79	36	9
March 21	128	621	434	145	113	72	367	164	137	234	94	36	9
March 22	158	904	434	196	113	72	506	164	137	318	94	36	9
March 23	225	904	632	196	117	72	532	251	245	318	135	70	50

*: Cumulative case incidences in the indicated countries were recorded from March 1st to March 23rd, 2020.

Supplemental online Table 2: COVID-19 in Middle-East, Africa, and Gulf countries.

	Iran	Pakistan	Saudi Arabia	Qatar	Bahrain	Egypt	Lebanon	Iraq	Kuwait	UAE	Morocco	Jordan	Tunisia	Oman	Afghanistan	South Africa	Algeria	Burkina Faso	Senegal	DRC	Cameroon
March 1*	593	4		1	40	1	2	13	45	19				6	1						
March 2	978	4		3	47	2	10	19	56	21				6	1						
March 3	1501	5	1	7	49	2	13	26	56	21	1	1	1	6	1		5				
March 4	2336	5	1	8	49	2	13	31	56	27	1	1	1	12	1		5				
March 5	2922	5	2	8	49	2	13	36	58	27	2	1	1	15	1		12				
March 6	3513	5	8	8	49	3	16	36	58	27	2	1	1	16	1		12		4		1
March 7	4747	5	8	11	49	3	22	44	58	45	2	1	1	16	1	1	17		4		2
March 8	5823	5	7	12	56	48	28	54	62	45	2	1	1	16	4	2	17		4		2
March 9	6566	6	15	15	79	55	32	60	64	45	2	1	2	16	4	3	20		4		2
March 10	7161	16	15	18	109	59	41	61	65	59	2	1	2	18	4	7	20		4		2
March 11	8042	16	20	24	110	59	41	61	69	74	3	1	6	18	4	7	20	2	4	1	2
March 12	9000	19	21	262	189	67	66	70	80	74	5	1	6	18	7	13	25	2	4	1	2
March 13	10075	20	21	262	195	67	66	70	80	85	6	1	7	18	7	17	25	2	10	1	2
March 14	11364	21	62	262	210	93	77	93	100	85	7	1	16	19	7	17	26	2	10	2	2
March 15	12729	28	103	337	211	93	93	93	112	85	18	1	16	20	10	38	37	3	21	2	3
March 16	14991	52	103	401	221	126	99	124	112	98	28	6	18	22	16	51	49	3	26	2	3
March 17	14991	187	133	439	229	166	109	124	130	98	38	35	24	21	21	62	60	15	27	3	5
March 18	16169	187	171	442	237	166	120	154	130	98	38	35	24	24	22	62	60	20	27	3	5
March 19	17361	241	238	442	258	196	133	164	142	113	49	52	29	33	22	116	72	26	36	7	10
March 20	18407	302	238	452	269	210	149	177	148	140	61	56	39	39	22	150	82	40	38	14	15
March 21	19644	461	274	460	285	256	163	193	159	140	74	69	54	48	24	205	94	40	38	14	22
March 22	20610	495	392	470	306	285	206	214	176	153	86	84	60	52	24	240	94	72	56	23	27
March 23	21638	748	511	494	337	327	248	233	189	153	115	112	75	55	40	274	201	75	67	30	40

*: Cumulative case incidences in the indicated countries were recorded from March 1st to March 23rd, 2020. UAE: United Arab Emirates. DRC: Democratic Republic of the Congo.

Supplemental online Table 3. Doubling times of COVID-19 cases in Italy by province *.

	Doubling	Geographical
AGRIGENTO	6.67	South
ALESSANDRIA	5.55	North
ANCONA	6.84	Center
AOSTA	6.29	North
AREZZO	5.86	Center
ASCOLI PICENO	5.19	Center
ASTI	5.41	North
AVELLINO	6.06	South
BARI	4.90	South
BAT	3.97	South
BELLUNO	6.10	North
BENEVENTO	6.25	South
BERGAMO	9.65	North
BIELLA	5.17	North
BOLOGNA	4.97	North
BOLZANO	6.12	North
BRESCIA	8.00	North
BRINDISI	7.54	South
CAGLIARI	6.55	South
CALTANISSETTA	4.50	South
CAMPOBASSO	6.33	Center
CASERTA	6.40	South
CATANIA	5.73	South
CATANZARO	6.21	South
CHIETI	5.64	Center
COMO	5.64	North
COSENZA	4.10	South
CREMONA	10.94	North
CROTONE	6.22	South
CUNEO	5.01	North
ENNA	1.95	South
FERMO	5.00	Center
FERRARA	5.00	North
FIRENZE	5.89	Center
FOGGIA	5.84	South
FORLCESENA	6.00	North
FROSINONE	4.86	Center
GENOVA	6.88	North
GORIZIA	4.69	North
GROSSETO	6.74	Center
IMPERIA	7.50	North
ISERNIA	6.67	Center
LAQUILA	4.50	Center
LASPEZIA	7.81	North
LATINA	5.17	Center
LECCE	5.35	South
LECCO	5.95	North
LIVORNO	5.50	Center
LODI	15.60	North
LUCCA	6.48	Center
MACERATA	5.60	Center
MANTOVA	6.39	North
MASSA-CARRARA	7.33	Center
MATERA	2.00	South
MESSINA	3.60	South
MILANO	6.38	North
MODENA	5.00	North
MONZA (BRIANZA)	5.07	North
NAPOLI	6.45	South

NOVARA	5.33	North
NUORO	14.33	South
ORISTANO	5.50	South
PADOVA	7.63	North
PALERMO	2.74	South
PARMA	6.12	North
PAVIA	9.15	North
PERUGIA	5.42	Center
PESARO	9.81	Center
PESCARA	6.27	Center
PIACENZA	9.20	North
PISA	5.63	Center
PISTOIA	6.78	Center
PORDENONE	4.18	North
POTENZA	5.62	South
PRATO	5.21	Center
RAGUSA	0.85	South
RAVENNA	5.70	North
REGGIO EMILIA	4.90	North
RIETI	4.75	Center
RIMINI	7.07	North
ROMA	5.53	Center
ROVIGO	6.45	North
SALERNO	5.22	South
SASSARI	5.70	South
SAVONA	7.81	North
SIENA	5.63	Center
SIRACUSA	6.20	South
SONDRIO	6.16	North
TARANTO	5.50	South
TERAMO	4.31	Center
TERNI	6.25	Center
TORINO	5.29	North
TRAPANI	5.50	South
TRENTO	5.26	North
TREVISO	7.03	North
TRIESTE	7.83	North
UDINE	6.59	North
VARESE	8.00	North
VENEZIA	6.78	North
VERBANO- CUSIO-OSSOLA	5.35	North
VERCELLI	5.68	North
VERONA	6.57	North
VIBO VALENTIA	3.83	South
VICENZA	6.39	North
VITERBO	5.15	Center
*: COVID-19 doubling times were computed on a province by province basis from landmark dates.		

Supplemental online Table 4. Doubling times of COVID-19 cases in Scandinavia by county *.

Sweden				Norway	
	Doubling time				Doubling time
DALARNA	7.17			AGDER	13.06
GAVLEBORG	8.25			INNLANDET	11.70
HALLAND	12.00			MOREOGROSMDAL	13.61
JONKOPING	7.91			NORDLAND	10.17
OREBRO	6.86			OSLO	11.64
OSTERGOTLAND	8.70			ROGALAND	17.29
SKANE	23.92			TROMSOGF	11.98
SODERMANLAND	8.62			TRONDELAG	12.96
STOCKHOLM	9.64			VESTFOLDOGT	12.86
UPPSALA	8.19			VESTLAND	14.27
VASTERBOTTEN	6.79			VIKE	12.88
VASTMANDLAND	6.13				
VASTRAGOTALAND	8.00				

*: Landmark dates were the 9th of April for Sweden and the 7th of April for Norway.

Supplemental online Table 5. COVID-19 cumulative incidence by calendar date in France.

DATE	TOTAL CASES
25/02/20	13
26/02/20	18
27/02/20	38
28/02/20	57
29/02/20	100
01/03/20	130
02/03/20	191
03/03/20	212
04/03/20	285
05/03/20	423
06/03/20	613
07/03/20	949
08/03/20	1126
09/03/20	1412
10/03/20	1784
11/03/20	2281
12/03/20	2876
13/03/20	3661
14/03/20	4499
15/03/20	5423
16/03/20	6633
17/03/20	7730
18/03/20	9134
19/03/20	10995
20/03/20	12612
21/03/20	14459
22/03/20	16689
23/03/20	19856
24/03/20	22302
25/03/20	25233
26/03/20	29155
27/03/20	32964
28/03/20	37575
29/03/20	40174
30/03/20	44550
31/03/20	52128
01/04/20	56989
02/04/20	59105
03/04/20	64338
04/04/20	68605

Supplemental online Table 6. COVID-19 cumulative incidence by calendar date in Germany.

DATE	TOTAL CASES
24/02/20	16
25/02/20	18
26/02/20	21
27/02/20	26
28/02/20	53
29/02/20	66
01/03/20	117
02/03/20	150
03/03/20	188
04/03/20	240
05/03/20	349
06/03/20	534
07/03/20	684
08/03/20	847
09/03/20	1112
10/03/20	1460
11/03/20	1884
12/03/20	2369
13/03/20	3062
14/03/20	3795
15/03/20	4838
16/03/20	6012
17/03/20	7156
18/03/20	8198
19/03/20	10999
20/03/20	13957
21/03/20	16662
22/03/20	18610
23/03/20	22672
24/03/20	27436
25/03/20	31554
26/03/20	36508
27/03/20	42288
28/03/20	48582
29/03/20	52547
30/03/20	57298
31/03/20	61913
01/04/20	67366
02/04/20	73522

Supplemental online Table 7. COVID-19 cumulative incidence by calendar date in UK.

DATE	TOTAL CASES
01/02/20	2
02/02/20	2
03/02/20	2
04/02/20	2
05/02/20	2
06/02/20	3
07/02/20	3
08/02/20	3
09/02/20	4
10/02/20	8
11/02/20	8
12/02/20	8
13/02/20	9
14/02/20	9
15/02/20	9
16/02/20	9
17/02/20	9
18/02/20	9
19/02/20	9
20/02/20	9
21/02/20	9
22/02/20	9
23/02/20	9
24/02/20	13
25/02/20	13
26/02/20	13
27/02/20	13
28/02/20	19
29/02/20	23
01/03/20	35
02/03/20	40
03/03/20	51
04/03/20	85
05/03/20	114
06/03/20	160
07/03/20	206
08/03/20	271
09/03/20	321
10/03/20	373
11/03/20	456
12/03/20	590
13/03/20	797
14/03/20	1061
15/03/20	1391
16/03/20	1543
17/03/20	1950
18/03/20	2626
19/03/20	3269
20/03/20	3983

21/03/20	5018
22/03/20	5683
23/03/20	6650
24/03/20	8077
25/03/20	9529
26/03/20	11658
27/03/20	14548
28/03/20	17104
29/03/20	19606
30/03/20	22271
31/03/20	25521
01/04/20	30088
02/04/20	34610
03/04/20	39282
04/04/20	43282
05/04/20	49481
06/04/20	53624
07/04/20	57512
08/04/20	63377
09/04/20	68052
